# Reinforcement Learning of Linking and Tracing Contours in Recurrent Neural Networks

**DOI:** 10.1371/journal.pcbi.1004489

**Published:** 2015-10-23

**Authors:** Tobias Brosch, Heiko Neumann, Pieter R. Roelfsema

**Affiliations:** 1 University of Ulm, Institute of Neural Information Processing, Ulm, Germany; 2 Department of Vision & Cognition, Netherlands Institute for Neuroscience (KNAW), Amsterdam, The Netherlands; 3 Department of Integrative Neurophysiology, Center for Neurogenomics and Cognitive Research, VU University, Amsterdam, The Netherlands; 4 Psychiatry Department, Academic Medical Center, Amsterdam, The Netherlands; University of Tübingen and Max Planck Institute for Biologial Cybernetics, GERMANY

## Abstract

The processing of a visual stimulus can be subdivided into a number of stages. Upon stimulus presentation there is an early phase of feedforward processing where the visual information is propagated from lower to higher visual areas for the extraction of basic and complex stimulus features. This is followed by a later phase where horizontal connections within areas and feedback connections from higher areas back to lower areas come into play. In this later phase, image elements that are behaviorally relevant are grouped by Gestalt grouping rules and are labeled in the cortex with enhanced neuronal activity (object-based attention in psychology). Recent neurophysiological studies revealed that reward-based learning influences these recurrent grouping processes, but it is not well understood how rewards train recurrent circuits for perceptual organization. This paper examines the mechanisms for reward-based learning of new grouping rules. We derive a learning rule that can explain how rewards influence the information flow through feedforward, horizontal and feedback connections. We illustrate the efficiency with two tasks that have been used to study the neuronal correlates of perceptual organization in early visual cortex. The first task is called contour-integration and demands the integration of collinear contour elements into an elongated curve. We show how reward-based learning causes an enhancement of the representation of the to-be-grouped elements at early levels of a recurrent neural network, just as is observed in the visual cortex of monkeys. The second task is curve-tracing where the aim is to determine the endpoint of an elongated curve composed of connected image elements. If trained with the new learning rule, neural networks learn to propagate enhanced activity over the curve, in accordance with neurophysiological data. We close the paper with a number of model predictions that can be tested in future neurophysiological and computational studies.

## Author Summary

Our experience with the visual world allows us to group image elements that belong to the same perceptual object and to segregate them from other objects and the background. If subjects learn to group contour elements, this experience influences neuronal activity in early visual cortical areas, including the primary visual cortex (V1). Learning presumably depends on alterations in the pattern of connections within and between areas of the visual cortex. However, the processes that control changes in connectivity are not well understood. Here we present the first computational model that can train a neural network to integrate collinear contour elements into elongated curves and to trace a curve through the visual field. The new learning algorithm trains fully recurrent neural networks, provided the connectivity causes the networks to reach a stable state. The model reproduces the behavioral performance of monkeys trained in these tasks and explains the patterns of neuronal activity in the visual cortex that emerge during learning, which is remarkable because the only feedback for the model is a reward for successful trials. We discuss a number of the model predictions that can be tested in future neuroscientific work.

## Introduction

**Fig 1:**
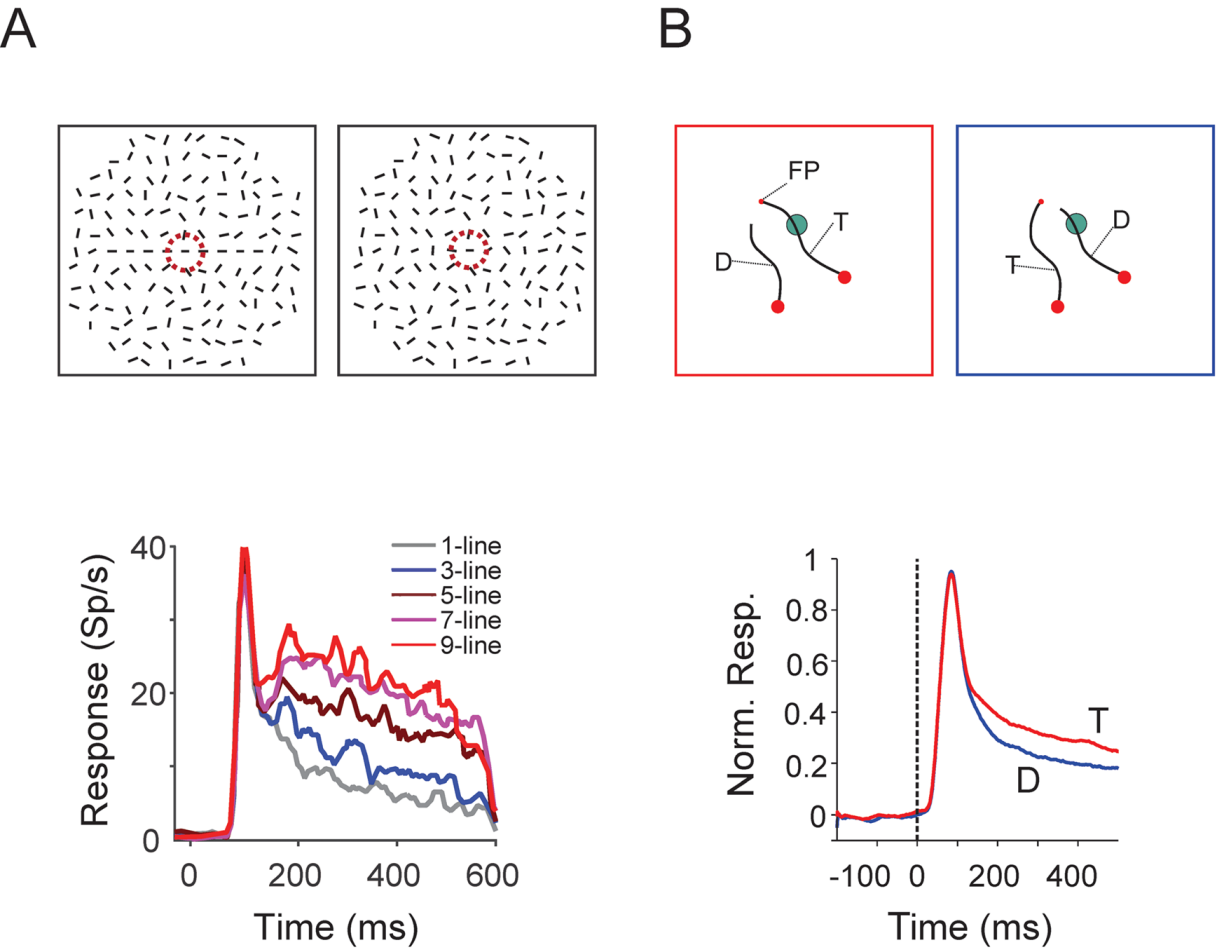
Neuronal correlates of contour integration and curve tracing in primary visual cortex (area V1). A) Contour integration task. If monkeys have been trained to make a saccade to a pattern with a string of collinear contour elements (1, 3, 5, 7 or 9 collinear bars, left panel), the neuronal responses in V1 elicited by these elements are stronger than the responses elicited by a single line element that is not part of such a perceptual group (right panel). This influence of colinearity on V1 activity is not present before training. The purple circle in the upper panel illustrates the V1 receptive field. Re-drawn from [15]. **B)** Curve-tracing task. Monkeys were trained to mentally trace a target curve (T) that is connected to a fixation point (FP) because they had to make an eye movement to a larger red circle at the end of this curve. They had to ignore a distractor curve (D). After training in this task, V1 activity elicited by the target curve (red response in lower panel) became stronger than that elicited by the distractor (blue response). The green circle in the upper panel shows the V1 receptive field. Adapted from [17].

Introspectively, visual perception appears to be remarkable effortless and automatic. Our perceptual world is filled with familiar objects and we do not experience many difficulties in judging where an object ends and the next one begins. The quality of image segmentation by the human brain surpasses segmentation in computer vision, which is known to be a hard problem [1], yet the precise mechanisms responsible for image segmentation in humans are only partially understood. In the present study we aim to explore the mechanisms that allow a neural network to learn to segment task-relevant image elements from a background of irrelevant elements. Image processing in humans and non-human primates can be subdivided in at least two phases. Research suggests that for many tasks the first phase is dominated by feedforward processing [2–5] (see [6, 7] for evidence for recurrent interactions in early vision). When a new image is presented to the visual system, information is rapidly propagated from early to higher visual areas. In this phase, the visual system extracts many elementary features such as colors, local orientations, contrasts, motion directions in low level areas and more complex features such as shape properties (curvature, corners) in higher areas [8]. This early processing phase thereby produces a pattern of activity across the various areas of the visual cortex that has been called “base representation” [8]. This early representation even includes certain object-categories such as animals or vehicles, which are detected very soon after the image has been presented [9, 10]. However, there are also many aspects of visual processing that require a more elaborate analysis than can be achieved during the first feedforward processing phase [4, 11]. Some tasks, such as image segmentation and contour grouping, demand the evaluation of the relations between items (spatial as well as temporal). Perceptual grouping and segmentation processes depend on a later serial processing phase where lateral connections between neurons in the same area and feedback connections that propagate information from higher areas back to lower areas come into play [2–5, 12, 13]. In this second processing phase, the activity of neurons in lower areas of the visual cortex is not only determined by the visual information in the neurons’ receptive field itself, but there is an additional influence of the context given by the activity of other neurons in the same area as well as representations in higher visual areas that provide feedback [11]. An example task where lateral and feedback connections play a role is contour grouping. For example, if monkeys are trained to detect a string of collinear contour elements [14], the neuronal responses in the primary visual cortex elicited by these elements are stronger than the responses elicited by line elements that are not part of such a perceptual group (Fig 1A; c.f. [14–16]). Interestingly, this contextual effect even occurs if the information in the V1 receptive field is held constant, which implies that it depends on the lateral influences of V1 neurons with different receptive fields and/or on feedback from higher visual areas where receptive fields are larger [8]. In accordance with this view, the effect of grouping on V1 activity does not occur during the initial visual response but at an additional delay (Fig 1A).

Another contour grouping task that is thought to rely on feedback and lateral connectivity is curve-tracing (Fig 1B) [8, 17–19]. In one version of this task, monkeys had to determine the endpoint of a target curve that started at the fixation point (“FP” in Fig 1B) as target for an eye movement, while ignoring another, distractor curve that was not connected to the fixation point. Also in this task, the contour elements that belong to the target curve were labeled in the visual cortex with enhanced spiking activity, and again, only after a delay relative to the initial visual response. The labeling of image elements with enhanced neuronal activity for grouping them into a coherent representation has been called *incremental grouping* [8]. The delayed response modulation reflects the monkeys’ belief about the stimulus [20]. It is much weaker if the monkey fails to perceive the perceptual group [15]. Furthermore, if the monkey groups the wrong set of image elements in his perception, this erroneous set of contour elements is labeled with enhanced activity [17, 21].

A number of previous modeling studies have investigated how feedforward, lateral and feedback connections determine the response of visual cortical neurons in these contour grouping tasks [22–25]. A key question that remains to be addressed, however, is how these grouping operations are learned during perceptual experience, because visual experience improves the detection and integration of image features [26–31]. That visual experience aids in image segmentation and perceptual grouping also follows from the fact that these processes are more efficient when objects are presented in their familiar orientations than when they are shown upside down [32, 33]. Perceptual learning in the contour integration task (Fig 1A) has been well documented in previous work. In this task, two weeks of experience greatly improve the accuracy of monkeys in detecting collinear image elements. Importantly, this training also increases the strength of the neuronal response modulation in V1 [14, 16]. During learning, the only feedback that the monkeys receive about their performance is a reward when they correctly detect the string of collinear contour elements and the omission of a reward when they fail to make the appropriate response. However, the neuronal mechanisms that underlie these reward-based perceptual grouping improvements are not well understood. How does the visual brain change connections between the appropriate neurons when feedback about performance is so limited?

In the present study we build upon previous models called AGREL (Attention-Gated Reinforcement Learning) and AuGMEnT (Attention-Gated MEmory Tagging) that have been proposed for the learning of feedforward connections from lower to higher areas in various cognitive tasks [34–36] and extend it to recurrent neural networks. These previous learning models proposed a three-stage mechanism for the adaptation of synaptic weights. In a first step, feedforward processing determines a winning unit in the output layer of the network that encodes the chosen action. In a second step, an attentional feedback (AFB) signal originating from the winning unit assigns credit to those connections that were responsible for the chosen action by creating synaptic tags. In the third step, a global learning signal determines the changes of the weights of those synapses that carry the plasticity tag. This global learning signal presumably corresponds to a neuromodulator such as dopamine, serotonin or acetylcholine that encodes the reward-prediction error, i.e. the difference between the amount of reward that was expected and the amount of reward that was actually received by the network [37–39]. These previous models only used attentional feedback signals originating from the output layer (step two) to highlight task relevant synapses, but did not use feedback or lateral connections for the labeling of image elements that belong to the same perceptual group with an enhancement of neuronal firing rates (as in Fig 1). Thus the mechanisms that permit reward-based learning in perceptual grouping tasks that rely on activity propagation through feedback and lateral connections have remained relatively unexplored.

To address learning in tasks with recurrent networks that utilize feedforward, lateral and feedback connections we will here propose a new learning scheme called RELEARNN (REinforcement LEArning in Recurrent Neural Networks) where the activity of the network depends in a complex manner on the visual input pattern, as recurrent connections allow the recirculation of activity. We will illustrate the capabilities of the new learning algorithm with the two contour grouping tasks of Fig 1, namely contour linking and curve tracing [14, 40]. We tested if RELEARNN can train the same neural network layout (and with the same parameters) to perform either task if the only feedback is a reward for correct performance and the omission of a reward in case of an error. We also investigated whether these networks develop coding strategies that resemble those in the visual cortex, where image elements of the same perceptual group are labeled with enhanced neuronal activity, and if and how these groups can be read out by higher areas to support the selection of the appropriate behavioral response. Our results demonstrate that (1) the behavior of the neural networks during learning and the pattern of errors is similar to that of monkeys that are trained in these tasks [14, 40], (2) the model reproduces the changes in neuronal firing rates and the emergence of incremental grouping during the learning process, (3) that the labeling with enhanced neuronal activity for grouping is an efficient code that can be used to guide behavior, and (4) that RELEARNN is a comprehensive and powerful learning scheme, which captures fundamental aspects of synaptic plasticity in a recurrent neural network. The model may thereby help to understand how neuromodulatory systems that code reward-prediction errors enable the learning of complex perceptual tasks that require the interactions between many units through feedforward, lateral and feedback connections. RELEARNN may represent the first *recurrent* neural network learning scheme that can explain the learning of both contour linking and curve tracing with a biologically plausible learning scheme.

In the following, we will first describe the proposed learning algorithm and the architecture of the neural network model that can be trained to perform the contour linking and curve tracing tasks. We will then present the simulations and compare them to the behavior and neuronal activity in monkeys trained on the same tasks, and will close with a comparison between RELEARNN and previous learning models.

## Models

We devised a novel learning algorithm to train recurrent networks by trial and error. In this section, we start by a description of the network model and its units. We will then describe the novel learning algorithm called “REinforcement LEArning in Recurrent Neural Networks” (RELEARNN).

### Model Units and Network Structure

**Fig 2:**
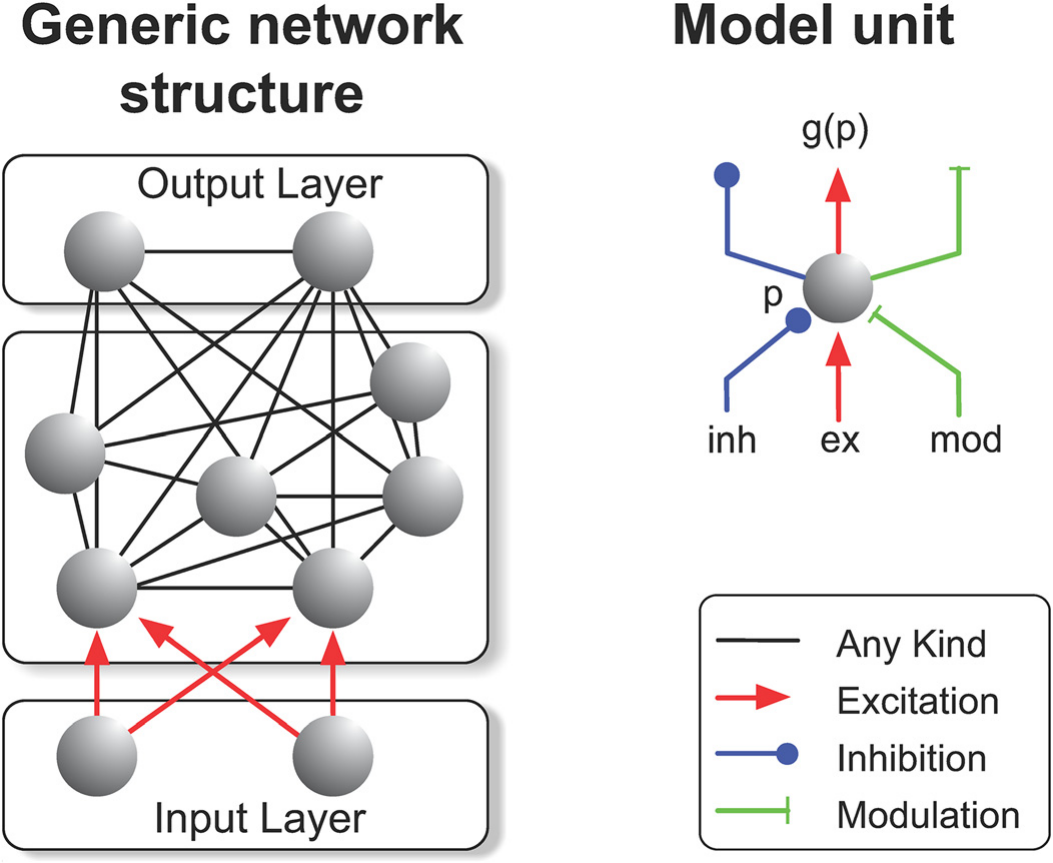
**Left**: Illustration of the network structure in its most general form, as used in the description of the learning algorithm. Every model unit *n*_*i*_ can excite, inhibit or modulate the activity of any other unit *n*_*j*_ (indicated by non-directional black connections). Units $n_{i}^{I}$ of the input layer provide input to the network but their activity does not depend on the activity of other units in the network. **Right**: A model unit (corresponding to a cortical column) can be excited, inhibited or modulated by other cortical columns.

The aim of the model is to compute the value of actions when it is presented with a visual stimulus. The model contains a number of output units (Fig 2), and it aims to approximate the value of each of the possible actions. These action values (known as *Q*-values [41]) are coded by the activity of the output units. The model usually chooses the action with the highest *Q*, but it will occasionally also explore other actions to promote learning. To find an appropriate balance between biological detail and mathematical tractability we chose model units with a scalar activation value, but to not include spiking neurons in our model. The model units represent the average activity in a cortical column with mean membrane potential *p* and mean firing rate *g*(*p*). As inputs, the model units receive excitation, inhibition as well as modulatory influences and the units, in turn, can inhibit, excite or modulate other model units. The role of the modulatory connections is to amplify the influence of excitatory connections, but these modulatory connections are unable to drive the units themselves (c.f. [42–46]). Note that modulatory connections have an effect that is synapse specific. Their role differs from the more global neuromodulatory signals like dopamine, acetylcholine or serotonine that are globally available in the network and gate plasticity. The model units used here have proven to be versatile building blocks of neural networks in previous studies (e.g. [47–56]). The membrane potential *p* depends on the excitatory, inhibitory and modulatory inputs *I* ^*ex*^, *I* ^*inh*^ and *I* ^*mod*^ as follows (Fig 2, right):

$$\begin{array}{c} \frac{d}{dt}p=-\alpha p+\left(\beta -p\right)\cdot I^{ex}\cdot \left(1+\gamma I^{mod}\right)-\left(\zeta +p\right)\cdot I^{inh}\;. \end{array}$$ (1)

The decay rate of the potential of model units is controlled by *α* > 0, the maximal potential by *β* > 0, the minimal potential by *ζ* > 0, and the parameter *γ* > 0 determines the impact of modulatory input. The mean spike rate *r* depends on the potential and is calculated as

$$\begin{array}{c} r=g\left(p\right)=\{\begin{array}{cc} a+p\;, & p\geq 0\;,\\ a\cdot \exp(p/a)\;, & p<0\;, \end{array} \end{array}$$ (2)

with *a* = 0.001.

Because the membrane potential is bounded by *β* (and −*ζ*), and because the network’s aim is to compute the action values as the activity *p* of the output units, we chose the reward of correctly performed trials below *β* and gave no reward if the model made an erroneous response.

We will now derive the learning algorithm, considering a network of *N* dynamically interacting model units with activities *p*_*i*_ receiving excitatory input ***I*** ^*inp*^ (see Fig 2 for the general structure of such a network, which may or may not be fully connected). Once the input has been given, the activity circulates through the excitatory, inhibitory and modulatory connections until the network activity stabilizes (convergence to a stable state is required for the current version of the learning algorithm). The overall dynamics are described by the following system of coupled differential equations (same as Eq (1), but now presented in vector notation)

$$\begin{array}{c} \frac{d}{dt}p=-\alpha p+\left(\beta -p\right)\cdot I^{ex}\cdot \left(1+\gamma I^{mod}\right)-\left(\zeta +p\right)\cdot I^{inh}\;. \end{array}$$ (3)

The excitatory, inhibitory and modulatory inputs ***I*** ^*ex*^,***I*** ^*inh*^ and ***I*** ^*mod*^ depend on the presynaptic firing rates and the input into the network ***I*** ^*inp*^

$$\begin{array}{c} I^{ex}={\left(W^{ex}\right)}^{T}\cdot g\left(p\right)+{\left(W^{inp}\right)}^{T}\cdot I^{inp}\;,\;I^{inh/mod}={\left(W^{inh/mod}\right)}^{T}\cdot g\left(p\right)\;. \end{array}$$ (4)

Here, *α*, *β*, *γ*, *ζ* ≥ 0 and *g* (applied element-wise) are defined as in Eq (1) and ***p***, ***I*** ^*inp*^ ∈ ℝ^*N*^ are column vectors of the activations and inputs of each unit. The positive elements $W_{kl}^{\left(\cdot \right)}\geq 0$ of the weight matrices ***W*** ^(⋅)^ ∈ ℝ^*N* × *N*^ determine the connection strength from unit *k* to unit *l* and $W_{kl}^{inp}$ determines the excitatory connection strength from feature *k* to input unit *l* (here products of two column vectors like ***p*** ⋅ ***I*** ∈ ℝ^*N*^ are defined element-wise). When the activity in the network has converged to a stable state (usually within a few hundred iterations), the network chooses one action based on the activation of the output units that encode the action (*Q*) values. We used the softmax rule to determine the probability *φ*_*a*_ of an output unit *a* to win the competition between all possible actions based on their values:

$$\begin{array}{c} \varphi_{a}=\frac{\exp(p_{a}/\tau )}{\sum_{j\in O}^{}\exp\left(p_{j}/\tau \right)}\;, \end{array}$$ (5)

where O is the set of all output units and *τ* is called a temperature parameter [41], which was adjusted to fit the experimental data (c.f. Suppl. A in S1 Text; adjusted by grid search). We here did not model how the softmax action selection process is implemented in the neural network, although this has been adressed in previous work [57]. Moreover, the choice of softmax as an action selection rule is not critical. We expect that other action selection mechanisms used in the reinforcement literature (e.g. *ɛ*-greedy [41] or max-Bolzman [58]) will give qualitatively similar results.

### Reinforcement Learning Algorithm for Recurrent Neural Networks (RELEARNN)

**Fig 3:**
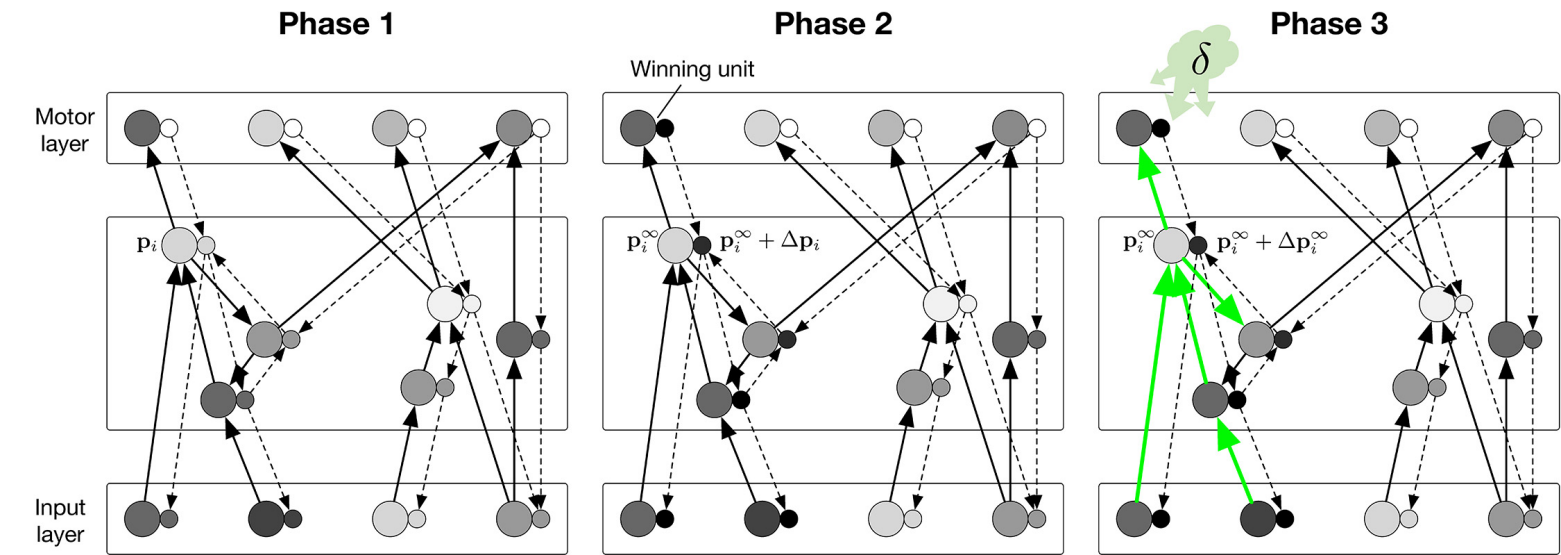
Illustration of the learning phases. Each regular unit (large circles) is accompanied by an accessory unit (small circles), which are hypothesized to be situated in the same cortical column. In phase 1, the sensory input leads to a stable state ***p*** ^∞^ of the regular units (note that we only illustrated the excitatory connections in this scheme) and the model represents estimates of the value of all the actions in the output layer. In phase 2, the winning output unit injects extra activity into the accessory network. The strength of the connections of the accessory network is reciprocal (equally strong) to that of the regular network. Accessory units that are paired with a regular unit that has a strong impact on the activity of the winning unit exhibit a strong increase in activity Δ***p*** during this phase. In phase 3 the changes in synaptic strength depend on Δ***p*** and a neuromodulatory signal that encodes the reward-prediction error *δ* (green cloud in phase 3; right panel).

Although research in past years has increased our knowledge about the mechanisms for the long-term modification of synaptic strength (e.g. [59, 60]), we will here focus on rather simple rules for synaptic modification. It is convenient to subdivide the mechanisms that lead to synaptic plasticity into a number of phases (c.f. [34, 35, 61]). Here we will distinguish between three phases. Phase one starts in response to the input and ends when the network converges to a stable state ***p*** ^∞^ and stochastically selects action *a* according to Eq (5). In the second phase, the selected output unit *a* causes an attentional feedback signal (AFB) that propagates through the network through a separate set of units (one per column; small circles in Fig 3) that change their response by Δ***p*** during this phase so that their total activity becomes ***p*** ^∞^ + Δ***p***. We call the network of units sensitive to the AFB the “accessory network” (see below for details), which is important for the guidance of synaptic plasticity.

Although many neurophysiological studies on the neuronal correlates of action selection targeted frontal and parietal cortex [62, 63], more recent studies reported that some neurons in the visual cortex are indeed also influenced by action selection, which is in accordance with the propagation of selection signals through an accessory network [64, 65]. More generally, there is evidence for a ubiquitous bi-directional “counter stream” interaction between higher and lower cortical regions [66–69] also justified by theoretical considerations (e.g. the selective tuning model for visual attention [70–72]). Furthermore, there is anatomical data to support a dichotomy of cortico-cortical connections. Studies from the lab of Sherman on the connectivity between areas of mouse cortex distinguished between class I and class II connections [73, 74]. Class I connections were called “drivers” because they are thought to activate neurons. They project from lower to higher areas but also in the opposite direction, from higher areas back to lower areas. Class II connections also project both in the feedforward and feedback direction, but they differ from class I connections because they are weaker and utilize the metabotropic glutamate receptor, involved in synaptic plasticity. It is therefore conceivable that the accessory network might employ class II connections, while the regular network employs class I connections, although this mapping is presently still speculative.

We will assume that the strength of the connections between units in the accessory network is similar (or proportional) to the strength of connections between the regular units (larger circles in Fig 3). This reciprocitity of regular and accessory connections is not a strong assumption because it can also be learned [34]. As we will demonstrate below, the boost in the membrane potential Δ*p*_*l*_ of the accessory unit *l* during the second phase is proportional to the influence of a change in *p*_*l*_ on the activity of the current winning unit $p_{a}^{\infty }$ during the first phase. Therefore, the AFB can be used to assign credit to those units that had an impact on the decision to take action *a*. Specifically, we will show that if an increase in the activity of unit *n*_*l*_ would increase the activity of the winning unit $p_{a}^{\infty }$ during the first phase, then Δ*p*_*l*_ > 0 during the second phase. If, on the other hand, unit *n*_*i*_ decreases $p_{a}^{\infty }$, this implies that Δ*p*_*l*_ < 0. The sign and magnitude of Δ*p*_*l*_ can be used to guide plasticity of synapses onto unit *l* if the aim is to decrease or increase $p_{a}^{\infty }$, i.e. to adjust the value of this action. Finally, in the third phase (c.f. Fig 3), the network receives a reward if it selects the correct action, but reward is omitted otherwise.

The output units of the network aim to represent the expected value if their action is chosen in the current sensory state (c.f. [35, 75]). Neurons in the frontal cortex, basal ganglia and midbrain are known to code for action values [76, 77], i.e. their activity appears to approximate the so-called *Q*-value, defined as the expected return *ϱ* when choosing action *a* in state *s* (c.f. [41]; note that rewards are often delayed; see discussion for extensions that deal with delayed rewards)

$$\begin{array}{c} Q_{a}=E_{\pi }\left\{\varrho |s,a\right\}\;. \end{array}$$ (6)

When the network performs action *a*, it receives a reward *ϱ* and the aim of the learning rule is to adjust the current estimate of *Q*_*a*_, represented by the activity of the winning output unit $p_{a}^{\infty }$. To this aim, the network computes a reward prediction error *δ* by comparing the outcome of the trial *ϱ* to the predicted *Q*-value, i.e. a SARSA style prediction error for immediately rewarded tasks [41]. In accordance with previous studies of reinforcement learning [78, 79], we assume that this reward prediction error is coded by a neuromodulatory signal that is globally released into the network so that it can influence the plasticity of all synapses (Fig 3, right panel).

$$\begin{array}{c} \delta =\varrho -Q_{a}=\varrho -p_{a}^{\infty }\;. \end{array}$$ (7)

Many dopamine neurons in the ventral tegmental area and substantia nigra encode the reward prediction error *δ* [37, 80, 81].

Once the network has received feedback about the chosen action *a*, the learning rule changes the connections of the network in order to decrease the reward prediction error for this action. Specifically, plasticity of the connection *w*_*kl*_ from unit *k* to unit *l* depends on four factors: (1) the presynaptic activity $r_{k}^{\infty }$, (2) the postsynaptic membrane potential $p_{l}^{\infty }$, (3) the activity of the accessory unit *l* $\Delta p_{l}^{\infty }$, which represents the influence of unit *l* on the activity of *a* and (4) the reward prediction error *δ*:

$$\begin{array}{c} \Delta W_{kl}=\eta \cdot \delta \cdot \Delta p_{l}^{\infty }\cdot f_{l}\left(p_{l}^{\infty }\right)\cdot r_{k}^{\infty }\;, \end{array}$$ (8)

where *η* is the learning rate. Note that the signals that determine plasticity are all available locally in the cortical column *l* and that Eq (8) implements a form of Hebbian plasticity, because it depends on the product of presynaptic activity $r_{k}^{\infty }$ and a function *f*(⋅) of the postsynaptic activity $p_{l}^{\infty }$. We will derive below that the form of *f*(⋅) differs between excitatory, inhibitory and modulatory connections projecting to column *l*:

$$\begin{array}{c} f_{l}^{ex}\left(p_{l}^{\infty }\right)=\left(\beta -p_{l}^{\infty }\right)\cdot \left(1+\gamma \cdot {\left(I_{\infty }^{mod}\right)}_{l}\right)\;, \end{array}$$ (9)

$$\begin{array}{c} f_{l}^{mod}\left(p_{l}^{\infty }\right)=\gamma \cdot \left(\beta -p_{l}^{\infty }\right)\cdot {\left(I_{\infty }^{ex}\right)}_{l}\;, \end{array}$$ (10)

$$\begin{array}{c} f_{l}^{inh}\left(p_{l}^{\infty }\right)=-\left(\zeta +p_{l}^{\infty }\right)\;. \end{array}$$ (11)

These equations follow from Eqs (1) and (3) and can be obtained by differentiating with respect to the corresponding input type (they represent the effective impact of the connection on the post-synaptic unit *l*).

### Credit Assignment by the Accessory Network

The aim of the accessory network is to determine $\Delta p_{l}^{\infty }$ for every unit *l*, which estimates the influence of *l* on the value of the selected action *a*. In the sequel we will capitalize on results of Almeida and Pineda [82, 83] who proposed a method to compute the error gradient in weight space for fully recurrent networks. It is based on the error-backpropagation rule, which computes the required changes in synaptic weights but it is thought to be biologically implausible [84, 85]. However, previous work on the AGREL [34] and AuGMEnT [35] learning rules demonstrated that in case of a reinforcement learning problem it is possible to replace the backpropagation of errors by two factors that are biologically plausible (reviewed by [86]): (1) an attentional feedback signal, which propagates activity rather than error signals from the output units to earlier processing levels, and (2) a globally released neuromodulatory signal that codes for the reward prediction error. We will here provide an equivalent result for the learning rule proposed by [82, 83] for fully recurrent networks.

Almeida [83, 87, 88] showed that once a fully recurrent network has settled in a stable state ***p*** ^∞^, the error gradient of all synapses can be computed by an accessory network if it is the transpose of the linearized original network. Thus, in the accessory network of [83] connections *W*_*kl*_ are replaced by connections $W_{lk}^{\prime }$ of equal strength but running in the opposite direction. His method injects the error signal in every output unit, i.e. a positive signal for output units that are not active enough and a negative signal for output units that are too active. The accessory network of Almeida then propagates the activity to units connected to the output units, and from there, successively to all other units in the network. In RELEARNN only the winning output unit *a* injects activity into the accessory network and this activity circulates until the accessory units reach a stable state.

#### Derivative of weight impact

We will now describe the application of the Almeida-Pineda algorithm [82, 87] to our network model. Our aim is to demonstrate that the activity of an accessory unit is proportional to the influence of the respective regular unit on the action value. We will first compute the influence of any synaptic weight *W*_*kl*_ on the unit coding the *Q*-value of the selected action *a*. To simplify the derivation, we will examine the propagation of signals in the accessory network under the assumption that the network contains only excitatory connections (for a full derivation using matrix calculus [89] including inhibitory and modulatory connections required to reproduce experimental findings see Suppl. C in S1 Text). In the episode when the regular network reaches its steady state, the change in the membrane potential of the units is governed by (recalling Eq (3), above):

$$\begin{array}{c} \frac{d}{dt}p=-\alpha p+\left(\beta -p\right)\cdot I^{ex}\cdot \left(1+\gamma I^{mod}\right)-\left(\zeta +p\right)\cdot I^{inh}\;. \end{array}$$ (3)

Under the assumption that there are no inhibitory and modulatory connections, this can be rewritten as

$$\begin{array}{c} \frac{d}{dt}p_{m}=-\alpha p_{m}+\left(\beta -p_{m}\right)\cdot \underset{=I_{m}}{\underset{︸}{\left(\sum_{n}W_{nm}\cdot g\left(p_{n}\right)+I_{m}^{inp}\right)}}\overset{!}{=}0\;,\;m=1\ldots N\;, \end{array}$$ (12)

which is zero at equilibrium. We can use this equation that governs the steady state to compute the influence of the synaptic weight *W*_*kl*_ on the activity of all units of the regular network, including the unit coding for the selected action. For simplicity, we omit the ∞ and just write ***p***, ***I*** instead of $p^{\infty },I_{\infty }^{ex}$. Computing the derivative (denoted by “′”) with respect to *W*_*kl*_ in Eq (12) results in

$$\begin{array}{c} 0=-\alpha \frac{\partial }{\partial W_{kl}}p_{m}-\overset{\text{product}\;\text{rule}\;{\left[\beta -p_{m}\right]}^{\prime }\cdot I_{m}+\left(\beta -p_{m}\right)\cdot I_{m}^{\prime }}{\overset{︷}{\overset{={\left[\beta -p_{m}\right]}^{\prime }\cdot I_{m}}{\overset{︷}{I_{m}\frac{\partial }{\partial W_{kl}}p_{m}}}+\left(\beta -p_{m}\right)\overset{=I_{m}^{\prime }}{\overset{︷}{\left(\delta_{lm}\cdot g\left(p_{k}\right)+\sum_{n}W_{nm}g^{\prime }\left(p_{n}\right)\frac{\partial }{\partial W_{kl}}p_{n}\right)}}}} \end{array}$$ (13)

$$\begin{array}{c} =\sum_{n}\underset{=:L_{mn}^{T}}{\underset{︸}{\left[-\delta_{nm}\cdot \left(\alpha +I_{m}\right)+\left(\beta -p_{m}\right)\cdot W_{nm}\cdot g^{\prime }\left(p_{n}\right)\right]}}\frac{\partial }{\partial W_{kl}}p_{n}+\left(\beta -p_{m}\right)\cdot \delta_{lm}\cdot g\left(p_{k}\right) \end{array}$$ (14)

$$\begin{array}{c} ={\left[L^{T}\cdot \frac{\partial }{\partial W_{kl}}p+e_{l}\cdot \left(\beta -p_{m}\right)\cdot g\left(p_{k}\right)\right]}_{m}\;, \end{array}$$ (15)

where (⋅)^*T*^ denotes the transpose of a matrix or vector, *δ*_*lm*_ = 1 if and only if *l* = = *m* (Kronecker delta), and the vector **e**_*l*_ ∈ ℝ^*N*^ is zero except for the *l*–th entry which is one, i.e.

$$\begin{array}{cc} \underset{=:L^{T}}{\underset{︸}{\left(\begin{array}{cccc} -\left(\alpha +I_{1}\right)+\left(\beta -p_{1}\right)W_{11}g^{\prime }\left(p_{1}\right) & \left(\beta -p_{1}\right)W_{21}g^{\prime }\left(p_{2}\right) & \ldots & \left(\beta -p_{1}\right)W_{N1}g^{\prime }\left(p_{N}\right)\\ \left(\beta -p_{2}\right)W_{12}g^{\prime }\left(p_{1}\right) & -\left(\alpha +I_{2}\right)+\left(\beta -p_{2}\right)W_{22}g^{\prime }\left(p_{2}\right) & & \left(\beta -p_{2}\right)W_{N2}g^{\prime }\left(p_{2}\right)\\ \vdots & \vdots & \ddots & \vdots \\ \left(\beta -p_{N}\right)W_{1N}g^{\prime }\left(p_{1}\right) & \left(\beta -p_{N}\right)W_{2N}g^{\prime }\left(p_{2}\right) & \ldots & -\left(\alpha +I_{N}\right)+\left(\beta -p_{N}\right)W_{NN}g^{\prime }\left(p_{N}\right) \end{array}\right)}}\cdot (\begin{array}{c} \frac{\partial }{\partial W_{kl}}p_{1}\\ \frac{\partial }{\partial W_{kl}}p_{2}\\ \vdots \\ \frac{\partial }{\partial W_{kl}}p_{N} \end{array}) & =(\begin{array}{c} 0\\ \vdots \\ -\left(\beta -p_{l}\right)g\left(p_{k}\right)\\ \vdots \\ 0 \end{array}) \end{array}$$ (16)

By multiplying this linear system with the matrix inverse ${L^{T}}^{-1}={L^{-1}}^{T}$, we obtain

$$\begin{array}{cc} \left(\begin{array}{c} \frac{\partial }{\partial W_{kl}}p_{1}\\ \frac{\partial }{\partial W_{kl}}p_{2}\\ \vdots \\ \frac{\partial }{\partial W_{kl}}p_{N} \end{array}\right) & ={L^{-1}}^{T}\cdot (\begin{array}{c} 0\\ \vdots \\ -\left(\beta -p_{l}\right)\cdot g\left(p_{k}\right)\\ \vdots \\ 0 \end{array})=(\begin{array}{c} -{\left(L^{-1}\right)}_{1l}^{T}\cdot \left(\beta -p_{l}\right)\cdot g\left(p_{k}\right)\\ -{\left(L^{-1}\right)}_{2l}^{T}\cdot \left(\beta -p_{l}\right)\cdot g\left(p_{k}\right)\\ \vdots \\ -{\left(L^{-1}\right)}_{Nl}^{T}\cdot \left(\beta -p_{l}\right)\cdot g\left(p_{k}\right) \end{array})\;. \end{array}$$ (17)

This equation describes the influence of *W*_*kl*_ on the activity of all network units. We are particularly interested in the influence $\frac{\partial }{\partial W_{kl}}p_{a}$ of *W*_*kl*_ on *p*_*a*_, i.e. the membrane potential of the unit that codes the value of the selected action *a*, which is given by

$$\begin{array}{c} \frac{\partial }{\partial W_{kl}}p_{a}=-{\left(L^{-1}\right)}_{al}^{T}\cdot \left(\beta -p_{l}\right)\cdot g\left(p_{k}\right)\;. \end{array}$$ (18)

Note that the *only non-local impact* of the synaptic weight from unit *k* to *l* on *p*_*a*_ is determined by ${\left(L^{-1}\right)}_{al}^{T}$, which describes the impact of the postsynaptic unit *l* on the winning output unit *a*. We will now demonstrate that $-{\left(L^{-1}\right)}_{al}^{T}$ corresponds to the activation level $\Delta p_{l}^{\infty }$ in the accessory network.

#### Accessory network

The learning rule ensures that the connections of the accessory network are reciprocal to those of the (linearized) regular network. The accessory network is linear because accessory units do not influence the gains determined by the (non-linear) regular network. These gains are taken into account by the accessory network during the activity propagation but will be treated as constants.

Before we describe the activity propagation within the accessory network itself, we will first describe a version of the regular network that we will linearize around its fixed point. Our aim is to determine the influence of a change of the synaptic weight *W*_*kl*_ on the position of this fixed point. At equilibrium, the activity of the regular network, defined in Eq (12), can be reformulated from

$$\begin{array}{cc} \frac{d}{dt}p_{m} & =-\alpha p_{m}+\left(\beta -p_{m}\right)\cdot \underset{=I_{m}}{\underset{︸}{\left(\sum_{n}W_{nm}\cdot g\left(p_{n}\right)+I_{m}^{inp}\right)}}=:f\left(p_{m}\right) \end{array}$$ (12)

to the following linear form (using Taylor’s formula)

$$\begin{array}{cc} \frac{d}{dt}p_{m} & \approx \underset{=0}{\underset{︸}{f(p_{m}^{\infty })}}+{\left[(\begin{array}{cccc} \frac{\partial }{\partial p_{1}}f\left(p_{1}^{\infty }\right) & \frac{\partial }{\partial p_{2}}f\left(p_{1}^{\infty }\right) & \ldots & \frac{\partial }{\partial p_{N}}f\left(p_{1}^{\infty }\right)\\ \frac{\partial }{\partial p_{1}}f\left(p_{2}^{\infty }\right) & \frac{\partial }{\partial p_{2}}f\left(p_{2}^{\infty }\right) & \ldots & \frac{\partial }{\partial p_{N}}f\left(p_{2}^{\infty }\right)\\ \vdots & \vdots & \ddots & \vdots \\ \frac{\partial }{\partial p_{1}}f\left(p_{N}^{\infty }\right) & \frac{\partial }{\partial p_{2}}f\left(p_{N}^{\infty }\right) & \ldots & \frac{\partial }{\partial p_{N}}f\left(p_{N}^{\infty }\right) \end{array})\cdot (\begin{array}{c} {\tilde{p}}_{1}\\ {\tilde{p}}_{2}\\ \vdots \\ {\tilde{p}}_{N} \end{array})\right]}_{m} \end{array}$$ (19)

where ${\tilde{p}}_{i}$ is the deviation of unit *i* from its original value at equilibrium (see Suppl. B in S1 Text for the Taylor expansion of the full interaction). The terms in the matrix of Eq (19) are defined as follows

$$\begin{array}{ccc} \frac{\partial }{\partial p_{n}}f\left(p_{m}\right) & = & -\delta_{nm}\alpha +\frac{\partial }{\partial p_{n}}\left[\beta -p_{m}\right]\cdot I_{m}+\left(\beta -p_{m}\right)\cdot \frac{\partial }{\partial p_{n}}(\sum_{i}W_{im}\cdot g\left(p_{i}\right)+I_{m}^{inp}) \end{array}$$ (20)

$$\begin{array}{cc} = & -\delta_{nm}\cdot \alpha -\delta_{nm}\cdot I_{m}+\left(\beta -p_{m}\right)\cdot W_{nm}\cdot g^{\prime }\left(p_{n}\right)\;, \end{array}$$ (21)

Thus, Eq (19) describes the *linearized interaction* of the *regular network at equilibrium* and can be used to determine how a small perturbation influences the equilibrium state. The perturbations influence the equilibrium state as follows

$$\begin{array}{c} f\left(p_{m}\right)\approx \sum_{n}[-\delta_{nm}\cdot \left(\alpha +I_{m}\right)+\left(\beta -p_{m}\right)\cdot W_{nm}\cdot g^{\prime }\left(p_{n}\right)]\cdot {\tilde{p}}_{n} \end{array}$$ (22)

$$\begin{array}{c} =-\left(\alpha +I_{m}\right)\cdot {\tilde{p}}_{m}+\sum_{n}\underset{={\tilde{W}}_{nm}}{\underset{︸}{\left[\left(\beta -p_{m}\right)\cdot W_{nm}\cdot g^{\prime }\left(p_{n}\right)\right]}}\cdot {\tilde{p}}_{n} \end{array}$$ (23)

$$\begin{array}{c} =-\left(\alpha +I_{m}\right)\cdot {\tilde{p}}_{m}+\sum_{n}{\tilde{W}}_{nm}\cdot {\tilde{p}}_{n}\;. \end{array}$$ (24)

This last equation provides a convenient way to describe the linearized interaction of the *regular network*. Following [82, 83], the *accessory network* with activities Δ***p*** is reciprocal to the (linearized) regular network at equilibrium Eq (24), and it also takes the gain factors *g*′(*p*_*n*_) and (*β* − *p*_*m*_) in every column into account plus an injection of a unit activation at the winning unit *δ*_*am*_ (note the indices of $\tilde{W}$ in Eqs (24) and (25) that indicate the reciprocity of activation flow). The accessory network converges to its own stable state as follows:

$$\begin{array}{cc} \frac{d}{dt}\Delta p_{m} & =-\left(\alpha +I_{m}\right)\cdot \Delta p_{m}+\sum_{n}{\tilde{W}}_{mn}\cdot \Delta p_{n}+\delta_{am} \end{array}$$ (25)

$$\begin{array}{c} =-\left(\alpha +I_{m}\right)\cdot \Delta p_{m}+\sum_{n}[\left(\beta -p_{n}\right)\cdot W_{mn}\cdot g^{\prime }\left(p_{m}\right)]\cdot \Delta p_{n}+\delta_{am} \end{array}$$ (26)

$$\begin{array}{c} =\sum_{n}\underset{=:L_{mn}}{\underset{︸}{\left[-\delta_{mn}\cdot \left(\alpha +I_{n}\right)+\left(\beta -p_{n}\right)\cdot W_{mn}\cdot g^{\prime }\left(p_{m}\right)\right]}}\cdot \Delta p_{n}+\delta_{am}\;, \end{array}$$ (27)

a time derivative that becomes zero once the accessory network has settled in a stable state. Thus, the signal that propagates in the accessory network from unit *n* to unit *m* equals *W*_*mn*_ ⋅ [(*β* − *p*_*n*_) ⋅ Δ*p*_*n*_] and the influence of this signal depends on *g*′(*p*_*m*_), the gain of unit *m*.

To summarize Eq (27), the associate network at equilibrium interacts as following:

$$\begin{array}{c} \frac{d}{dt}\Delta p=L\cdot \Delta p+J=0\;, \end{array}$$ (28)

with ***J*** = 0 except for *J*_*a*_ = 1. At steady state (i.e. $\frac{d}{dt}\Delta \text{p}=0$), for the accessory network it holds (by multiplying Eq (28) with $L^{-1}$)

$$\begin{array}{c} \Delta p_{l}^{\infty }=-{\left(L^{-1}J\right)}_{l}=-\sum_{n=1}^{N}{\left(L^{-1}\right)}_{ln}J_{n}=-{\left(L^{-1}\right)}_{la}\;, \end{array}$$ (29)

i.e. $\Delta p_{l}^{\infty }$ can be plugged into Eq (18). This result demonstrates that by circulating the activity initiated by the winning action *a*, the activity Δ*p*_*k*_ of units of the accessory network becomes equal to the impact that the corresponding regular units have on the action-value of the winning unit *a*.

#### The activity of an accessory unit is proportional to the influence of the regular unit on the action value

With the use of Eqs (29), (18) can be written as

$$\begin{array}{cc} \frac{\partial }{\partial W_{kl}}p_{a} & ={\Delta p_{l}}^{\infty }\cdot \underset{=f_{l}\left(p_{l}\right)}{\underset{︸}{\left(\beta -p_{l}\right)}}\cdot \underset{=r_{k}}{\underset{︸}{g(p_{k})}}\;. \end{array}$$ (30)

Which proves that for an error term $E=\frac{1}{2}{\left(\varrho -p_{a}^{\infty }\right)}^{2}=\delta^{2}$ (describing the quadratic distance between the estimate $p_{a}^{\infty }$ and the experienced reward *ϱ*; c.f. Eq (7)) our learning rule Eq (8) performs gradient descent:

$$\begin{array}{c} \frac{dW_{kl}^{\left(\cdot \right)}}{dt}=-\eta \frac{\partial E}{\partial W_{kl}}=\eta \cdot \delta \cdot \frac{\partial p_{a}^{\infty }}{\partial W_{kl}}=\eta \cdot \delta \cdot {\Delta p_{l}}^{\infty }\cdot f_{l}\left(p_{l}\right)\cdot r_{k}\;. \end{array}$$ (31)

Thus, with the help of the accessory network the information necessary to shift the equilibrium state of the regular network in a direction that improves the estimate of the value of the chosen action becomes available locally, in the cortical column. To simplify the presentation in this section we focused on networks with only excitatory connections, but we also included inhibitory and modulatory connections in our full network. The generalization to networks that include modulatory and inhibitory connections is provided in Suppl. C in S1 Text.

## Results

**Fig 4:**
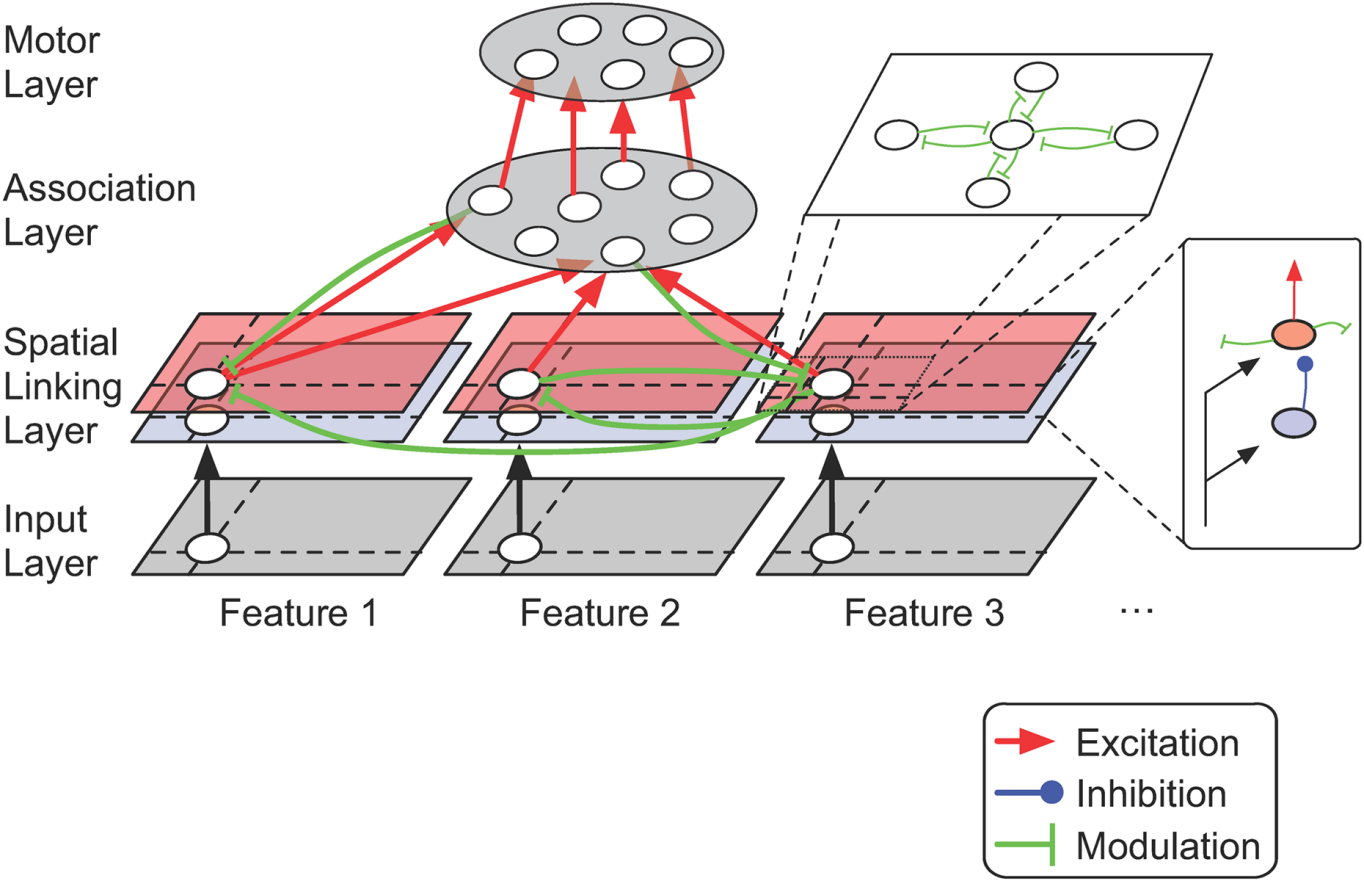
Neural network structure. The input layer contains 2D-maps of feature selective units (corresponding to representations in cortical area V1), which provide input to a “linking layer” that can establish perceptual groups. Input units activate excitatory and inhibitory units in the linking layer, and inhibitory units provide disynaptic inhibition to the excitatory units in this layer. Modulatory connections (green), which increase the excitatory impact, interconnect excitatory units with adjacent receptive fields in the same feature maps and units with overlapping receptive fields in different feature maps. Excitatory units in the linking layer can activate any unit in the association layer (e.g. in extrastriate or parietal cortex), and receive modulatory feedback connections. Units in the association layer, in turn, activate units in the motor layer (corresponding to neurons in the frontal eye field) that represent action-values and select one of a number of actions. Black connections have a fixed strength and excitatory (red), inhibitory (blue) and modulatory (green) connections undergo synaptic plasticity.

The analysis described above establishes that RELEARNN can train a fully recurrent network to adjust its fixed point and to thereby improve its estimate of the value of a selected action. To test the performance of the learning algorithm, we investigated if RELEARNN would train a neural network to group contour elements and to trace curves, two tasks thought to require feedback and horizontal connections in the brain. Will the network solve either task if the only feedback from the environment is a reward for correct responses? Although RELEARNN can train recurrent neural networks with many different structures, we focused on networks with a single achitecture that has been illustrated in Fig 4. Input units of the network represent the state of the environment and it is the task of the network to select a saccadic eye movement by activating one of the units in the motor layer, where units code for a range of possible eye movements. The learning algorithm tries to adapt the connection weights so that the activity of units in the motor layer code the expected reward for making a saccade to that location. The softmax rule for action selection ensures that saccades with a higher action value (higher activity of the motor unit) have a higher probability to be selected. We provide the detailed parameters for the simulations in Suppl. A in S1 Text. The model structure includes horizontal and feedback connections. Units in the input layer can activate excitatory units in the linking layer or provide disynaptic inhibition. Excitatory units in the linking layer can modulate the activity of their four neighbors that are selective for the same feature and they can also provide modulatory input to units at the same spatial location that are tuned to a different feature. Furthermore, units of the linking layer excite units in the association layer and they are the target of modulatory feedback connections from the association layer. Units in the association layer, in turn, excite units in the motor layer. Thus, the lateral connections and feedback connections from the association layer back to the linking layer enable the recirculation of activity through the network, i.e. recurrent processing. The structure of the input and motor layers differed between the contour-linking and curve-tracing tasks, because the inputs and the eye movement responses were different (described below). To keep the complexity of the models at a minimum, we did not include all connection types. For example, we omitted feedback connections from the motor units to the association layer as well as lateral and inhibitory connections within the motor layer. We found that the set of connections included in the model was rich enough to solve the two tasks. It is likely, however, that other tasks that we did not model might benefit from additional connection types.

### Learning to Link Visual Contours

Li, Piëch and Gilbert trained monkeys to group colinearly aligned contour elements using the task illustrated in Fig 1A [14]. In every trial, the monkey saw two sets of short line elements within circular apertures. One of the apertures contained a contour pattern composed of a number of colinear line elements and the other pattern contained line elements with a random orientation. The overall orientation of the colinear contour was fixed during a training session, but it could vary across sessions. At the start of a trial, the monkey had to direct gaze to the center of the display. The animals’ task was to make an eye movement to the aperture with the contour pattern (Fig 1) and the animals performed approximately 1500 trials per day. The authors started with a pretraining phase of several days where the target contour was shown at a high luminance but the distractor elements were less visible because they had a lower luminance. This was followed by the main training sessions where the distractor elements had the same luminance as the target elements so that only colinearity remained as a cue to solve the task. We investigated whether RELEARNN could train the network of Fig 4 by rewarding saccades to the contour pattern. Will the network learn to detect the colinearly aligned contour elements and will it reproduce the monkeys’ behavior during learning? Moreover, how does the activity of network units compare to the neuronal activity in the visual cortex of the monkeys [14]?

Li et al. trained their monkeys to maintain fixation for several hundreds of milliseconds upon presentation of the patterns and to make the saccade after this delay [14]. We trained the neural network on a version of the task that was simpler because we did not model the different phases of every trial. The model could immediately select the eye-movement upon presentation of the pattern and convergence to a stable activity state (learning of multiple task epochs by a feedforward network has been addressed by [35, 36]). The input to the network consisted of two patterns of bars with four possible orientations presented on a grid with 9 × 9 spatial positions on either side of the fixation point. There were a total of 648 input units (81 locations per pattern × 4 orientations × 2 patterns) that provided direct excitation and disynaptic inhibition via inhibitory units to the excitatory units of the linking layer, with 2 × 4 × 9 × 9 units, one for every orientation at each retinotopic location. Excitatory units in the linking layer projected to units with adjacent receptive fields tuned to the same orientation (four nearest neighbors) and also to the three units with the same receptive field tuned to other orientations with modulatory connections. Units of the linking layer propagated activity to a total of four units in the association layer, which, in turn, projected to the two units of the motor layer that had to learn the expected reward associated with a saccade to the left or right pattern. To facilitate learning, we started with a short (500 stimulus presentations) preliminary procedural training phase (just like [14]; this was not necessary, however, to successfully learn the task). During the pre-training phase, we made the background surrounding the contour elements less salient than the target pattern by reducing their contrast (activity of input units representing background elements was set to 50%). We then increased the contrast of the background contrast in increments of 10% (100 trials per contrast step) until all elements had 100% contrast.

**Fig 5:**
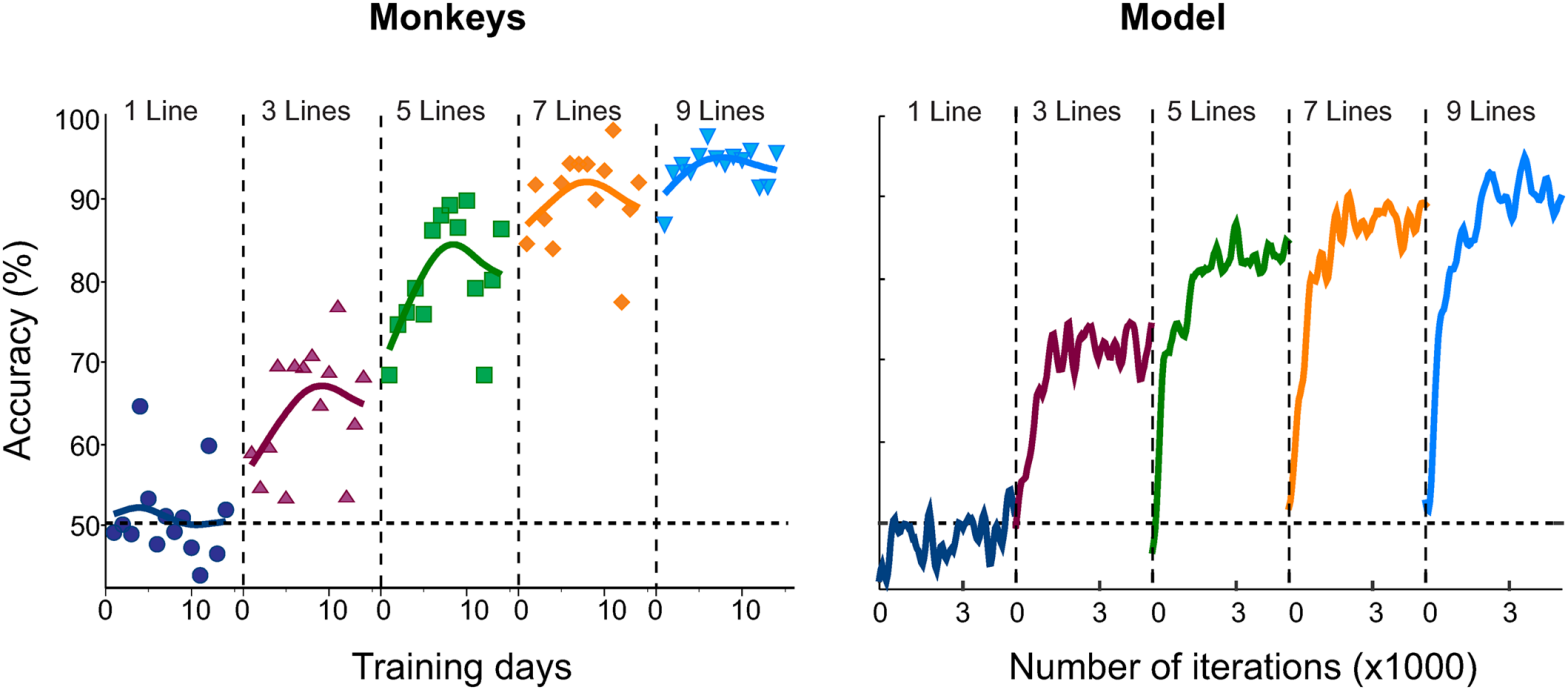
Accuracy of the model and comparison to the accuracy of monkeys. Left, performance of monkeys re-drawn from [14]. The five panels show the accuracy across training days with patterns of increasing contour length; 1 line (blue), 3 (purple), 5 (green), 7 (orange) or 9 colinear lines (light blue). Note that the monkeys performed at chance level for patterns with line length 1, which are indistinguishable from distractor patterns. Solid lines are cubic spline fits. **Right**, Accuracy of the model for the same stimuli, smoothed with a Gaussian (*σ* = 100 trials). The number of iterations refers to repetitions of the same contour length, i.e. the total number of iterations is five times as large because the model was exposed to five different contour lengths.

The pre-training phase was followed by training in the full task (Fig 5). Training caused a gradual increase in the model’s accuracy and the effect of training was particularly pronounced for patterns with a larger number of colinear line elements. The effect of training on the accuracy of the model resembled the improvement in the accuracy of the monkeys (c.f. Fig 5 left vs. right). The model was able to learn the contour linking task within ∼ 15,000 trials (∼ 3,000 trials per contour length), which is comparable to the time-course of the monkeys’ behavioral improvement (10 days with ∼ 1,500 trials per day). The results of these simulations were not critically dependent on the exact parameter values, i.e. slight parameter changes did not qualitatively alter the outcome, although less optimal values of the parameters decreased learning speed.

**Fig 6:**
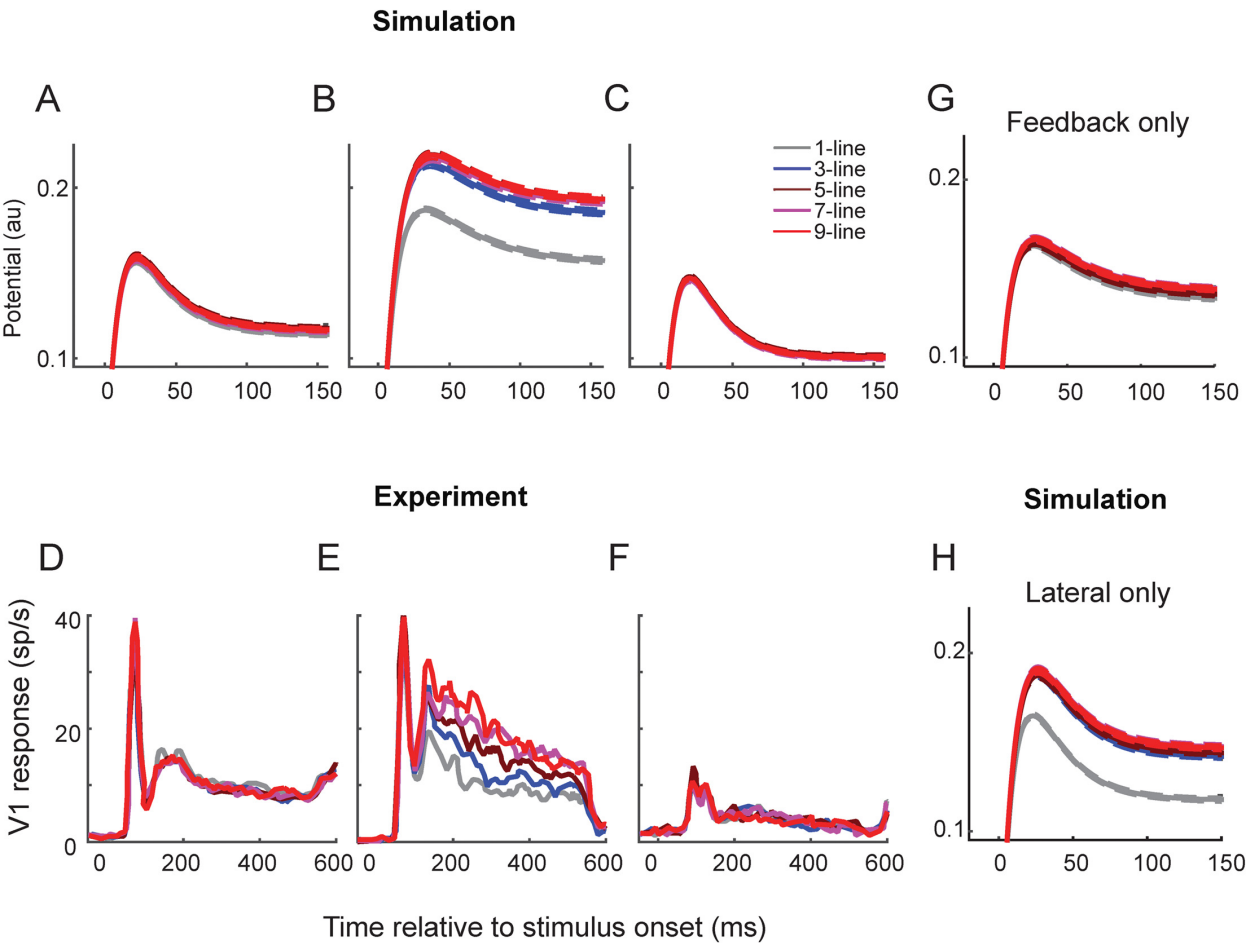
Comparison between the activity of model units and neurons in area V1 of monkeys. **A,B** Simulation results (ordinate in arbitrary units). Mean activity *p* (membrane potential) of excitatory units in the linking layer (red in Fig 4) representing the center bar of the target stimulus and the 95% confidence interval (across 10,000 stimulus presentations) before **(A)** and after training in the contour detection task **(B)**. **C**, Activity in a trained model when the strength of the modulatory horizontal and feedback connections is set to zero. **D,E**, Mean activity of V1 neurons elicited by the target stimulus before **(D)** and after training **(E)**. Training caused V1 neurons to be tuned to number of colinearly aligned bars. **F**, Under anesthesia, the sensitivity to contour length was abolished and the visual responses were reduced in strength but not abolished. **D,E,F** redrawn from [14]. **(G)** Activity in a model where the lateral connections in the linking layer were removed. **(H)** Activity in a network where feedback connections from the association layer to the linking layer were removed.

We next investigated how learning of the contour integration task changed the activity of network units (Fig 6). After the pre-training, but before the training in the full task, the activity of the excitatory units in the linking layer did not depend strongly on the number of colinearly aligned contours in the target pattern (Fig 6A). After training, however, the activity of these units clearly depended on the number of colinearly aligned contour elements (Fig 6B). Patterns with more colinearly aligned contour elements elicited stronger responses, thereby reproducing the effect of training in area V1 of monkeys (Fig 6D and 6E). It is also of interest to compare the time course of activity in the model’s linking layer to the time-course of activity in monkey V1. In the trained model, tuning to the number of colinear line elements was relatively weak during the initial response, but it became stronger when the network units started to converge to their stable activity level. The feedforward activation from the input layer only conveys information about the orientation of the contour element in the receptive field and not about the context provided by image elements in the surround. Thus, tuning to colinearity must be due to alterations in horizontal and feedback influences on the units, and this explains the delay in the emergence of this tuning. The temporal profile of the response of model units is quite comparable to that of V1 neurons, for which tuning to the colinearity of image elements also emerges during the later phase of the response.

In their experiment, Li et al. [14] also anesthetized the monkeys after the training procedure with the aim to block the top-down and horizontal influences. During anesthesia, the tuning to colinearity was indeed abolished in area V1, whereas visually driven activity was maintained, albeit weaker (Fig 6F). We could replicate the effect of anesthesia in our model by removing the feedback and lateral connections (Fig 6C), which confirmed that these contextual influences in the model are mediated by recurrent, i.e. lateral and feedback connections. In addition, we tested the respective contribution of lateral and feedback interactions by deleting one set of connections or the other (Fig 6G and 6H). The results indicate that lateral interactions have a more pronounced effect than feedback interactions in this task, a prediction that can be tested in future experiments.

**Fig 7:**
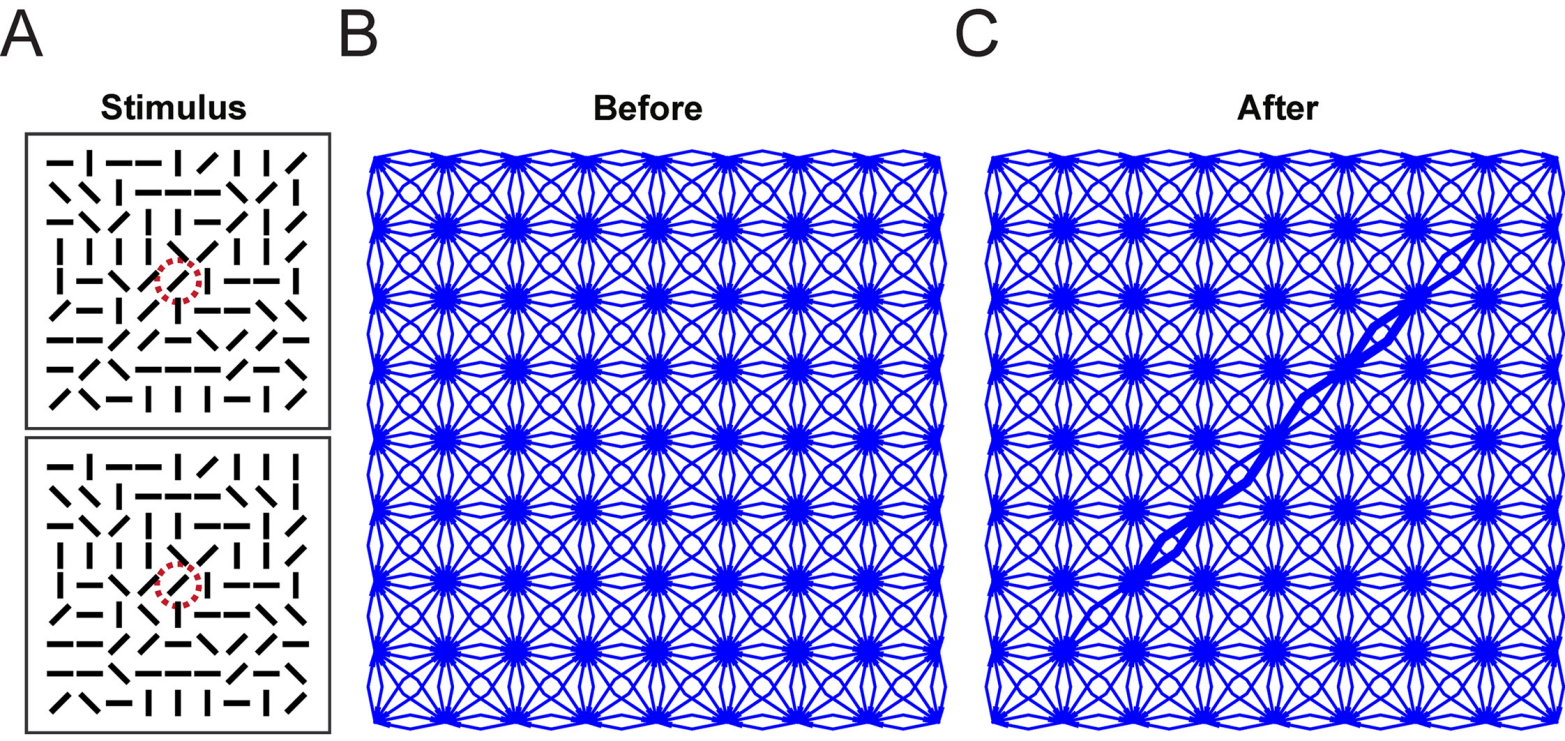
Stimulus and lateral connectivity in the “linking layer”. **A** Example stimulus (the patterns presented to the two hemispheres are plotted above each other). Re-drawn from [15] **B** Lateral connectivity in the “linking layer” for one orientation and hemisphere for units selective for diagonal line elements before training. **C** Lateral connectivity after training (we observed similar patterns for the other orientations). Line thickness corresponds to connection strength (thickest line corresponds to a connection strength of 0.36). Note that due to the small amount of simulated units (one unit per spatial input location) we did not introduce jitter into the stimulus which would have resulted in multiple parallel “thick lines”.

**Fig 8:**
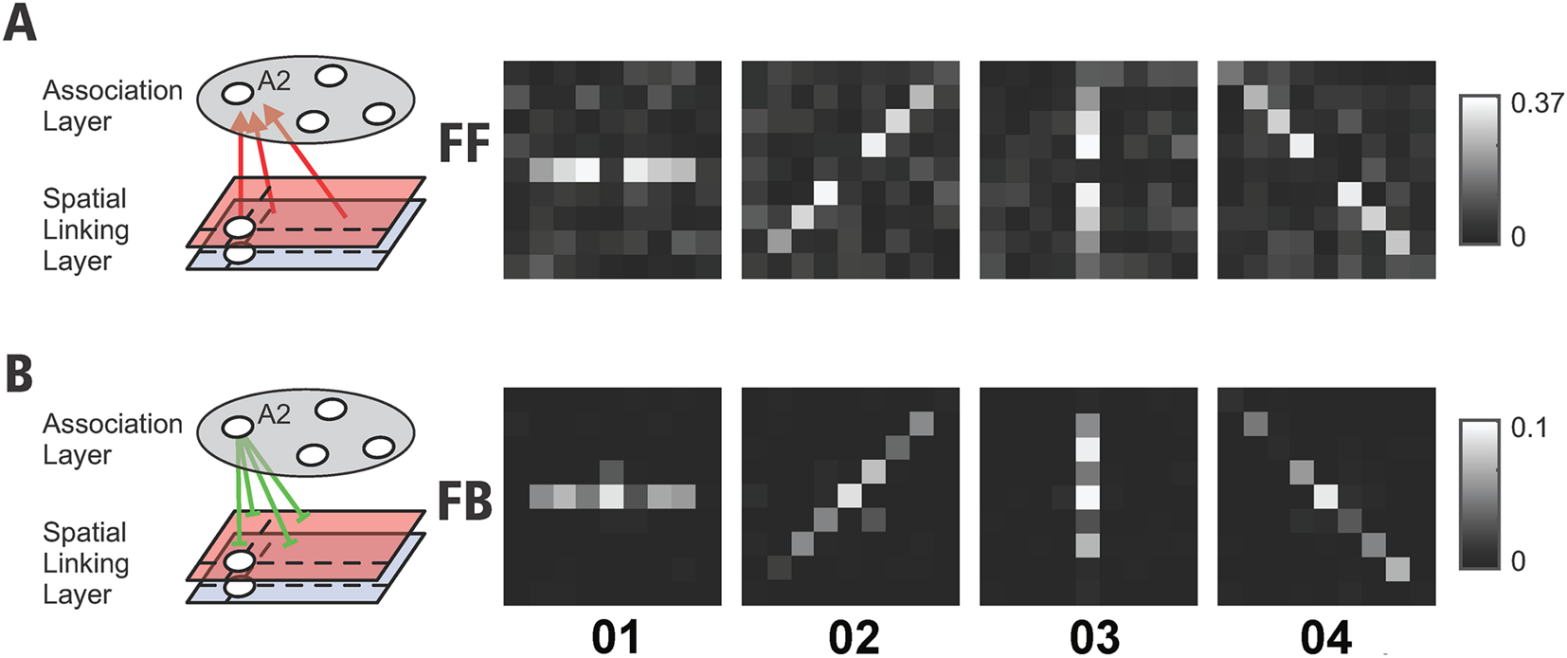
Connection strengths between linking units with different orientation preferences (O1–O4 denote the different orientations of contour elements) in *one* hemifield and an example association layer unit A2 with a strong connection to the motor unit in this hemisphere, after training to criterion (feedforward strength A and feedback strength B). Similar association units were established in the other hemisphere.

We next investigated how training in the contour linking task influenced the pattern of connectivity. We first examined the horizontal connections in the linking layer of an example network that had been trained to criterion (Fig 7). It can be seen that the lateral connections were strengthened along the direction corresponding to the axis of the unit’s orientation tuning. We next examined the pattern of excitatory feedforward connections from the linking layer to units in the association layer. In this analysis we focused on association units with a strong connection to motor units, which have an impact on the computation of the action values. We found that the association units integrated the activity of units along the target contours, with a specific set of connections from each of the four orientation maps (Fig 8A). When we examined the pattern of modulatory feedback connections from the association layer back to the linking layer we found that connections of the regular network had become largely reciprocal (in addition to the accessory network connections, which are enforced to be reciprocal). Association units tended to have strong feedback connections to units that provided them with feedforward excitation (Fig 8B). This reciprocity of feedforward and feedback connections is in accordance with anatomical findings [90, 91]. To summarize, the new RELEARNN algorithm reproduced the time-course of neuronal activity underlying contour linking as observed by Li et al. [14] and illustrates how it can be explained by a modification of the pattern of feedforward, lateral and feedback connections.

In our main simulations, units in the linking layer were only connected if they represented nearby contour elements. To examine if this restriction was necessary for successful learning we also carried out simulations with full connectivity in the linking layer. We found that networks with full connectivity also learned the task and, interestingly, strongest connections formed between units with adjacent receptive fields as the result of the training process.

We next examined whether recurrent connections were necessary for detection of the colinear contour configurations. We therefore trained networks without lateral connections in the linking layer and without feedback connections from the association layer to the linking layer (we kept the feedback connections of the accessory network that guide plasticity). Activity in these feedforward networks is immediately stable and it is therefore not necessary to wait until the network converges, so that RELEARNN becomes equivalent to the AuGMEnT learning rule for feedforward networks [36]. RELEARNN could train these purely feedforward networks to detect the colinear contours. This is an important result, which implies that the detection of these colinear contour patterns does not require recurrent connectivity. Nevertheless, networks with recurrent connections do utilize these connections if trained with RELEARNN, in accordance with the neurophysiological findings.

### Learning to Trace a Curve

**Fig 9:**
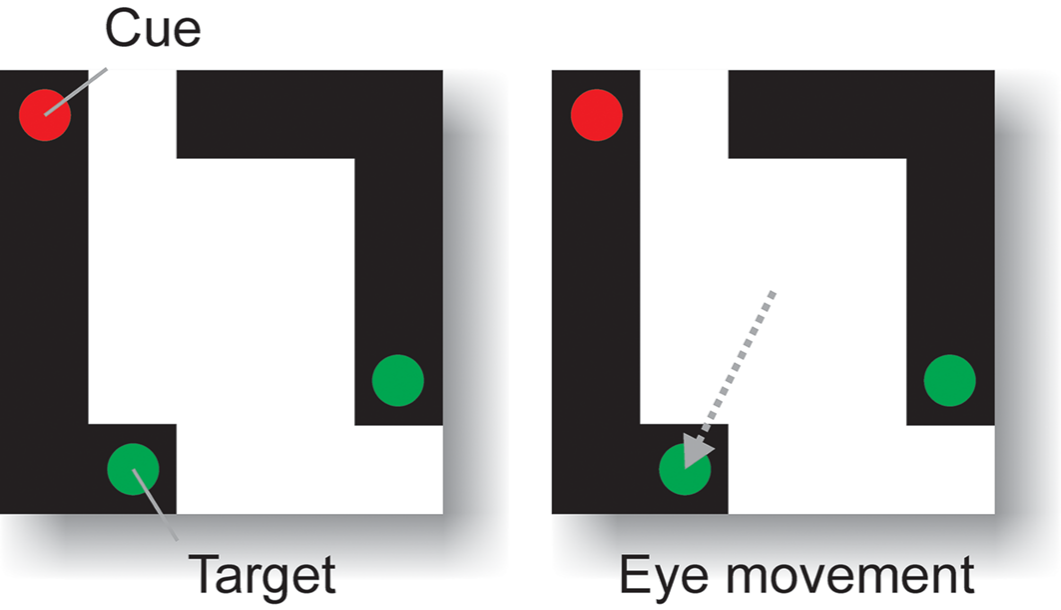
Curve tracing task. We trained the model to make an eye movement to a green circular marker that was on the target curve, which was cued with a red circle. The other curve was a distractor and had to be be ignored. This task requires the grouping of the connected image elements of the target curve.

**Fig 10:**
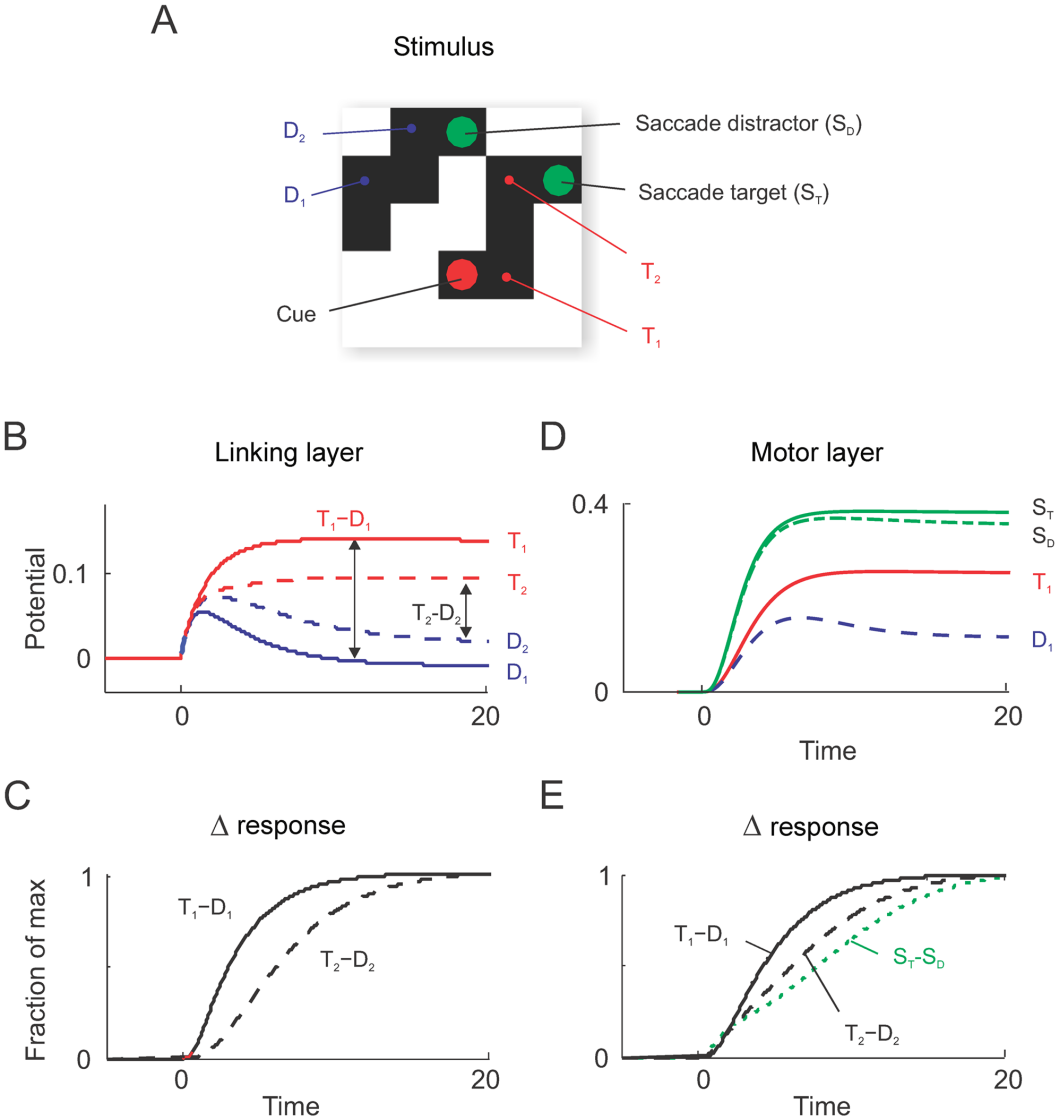
Activity in an example network trained in the curve-tracing task. A, An example stimulus with two green saccade targets and a red cue that indicates which curve is target. T1 and T2 are two pixels of the target curve and D1 and D2 are pixels of the distractor (we evaluated activity for different stimuli at the equivalent locations). **B**, Activity *p* (membrane potential) of example units of the linking layer, averaged across multiple stimulus presentations. The curve tracing task induced an increase of activity of units representing the target curve (T1 and T2) and a decrease in the activity of units representing the distractor (D1 and D2; 95% point-wise confidence bands are within line width). **C**, We normalized the difference in activity elicited by corresponding positions of the target and distractor curve in the linking layer to investigate the time-course of the response enhancement. We found that the latency of the response enhancement increased for pixels of the target curve that are farther from the red cue, in accordance with previous neurophysiological results [95]. **D**, Activity in the motor layer was strongest for pixels with a green cue. Note that the activity elicited by the saccade cue on the target curve (*S*_*T*_) was stronger than that elicited by the saccade cue on the distractor curve (*S*_*D*_). **E**, Time course of normalized response differences in the motor layer. Also here the response enhancement occurred later for pixels that were farther from the red cue. The activities in the motor layer **(E)** and the linking layer **(C)** have similar time-courses as have been reported in the frontal and visual cortex of monkeys [17]. Note that the propagation is quite fast due to the small network size but that it critically depends on the recurrent interaction as theoretically predicted by [96].

Not all conceivable feature groupings can be coded by dedicated neurons [8]. If confronted with a new configuration of image elements, grouping can proceed based on low level grouping criteria, such as connectedness or colinearity, even in the absence of neurons that are selective for the overall shape. In these situations, the visual brain appears to code perceptual groups by labeling the to-be-grouped image elements with enhanced neuronal activity, a process that has been called incremental grouping [8]. One example task that requires the formation of incremental perceptual groups is curve-tracing [18, 92] where subjects have to group contour elements that belong to a single elongated curve. Fig 9 shows one example stimulus of the curve tracing task that we used to test RELEARNN. Every stimulus contained two curves that consisted of connected pixels, two potential green eye movement targets and a red cue. The model’s task was to select an eye movement to a green marker on one of the two curves, which will be referred to as “target curve”. The target curve was cued with the red marker and the model was only rewarded if it made an eye movement to the green marker on the cued target object. Thus, the model had to learn to apply connectedness grouping to determine which of the two green markers fell on the same curve as the red cue. Human observers solve such a curve-tracing task by mentally tracing along the target curve. This mental tracing process corresponds to the gradual spread of object-based attention over the target curve [8, 93]. Previous neurophysiological studies revealed a neuronal correlate of the spread of object-based attention over a curve in the primary visual cortex because neuronal activity elicited by a target curve was stronger than that elicited by a distractor (Fig 1B). The response enhancement does not occur during the transient response that is triggered by the onset of the visual stimulus and the appearance of contour elements in the neurons’ receptive fields but after a delay of ∼ 150ms. The enhanced neuronal activity first occurs at the cued location (i.e. the red cue in Fig 9) before it gradually spreads over the other image elements of the same object [94, 95]. These results suggest that the enhanced neuronal activity spreads through horizontal connections in visual cortex, which link neurons that represent adjacent image elements, although it is likely that feedback connections from higher visual areas to the primary visual cortex also contribute. Similar effects have been observed in frontal cortex where the target curve also evoked stronger responses than the distractors during a delayed phase of the neuronal response [17, 40]. A previous modeling study investigated the propagation of enhanced activity along a relevant curve in a handcrafted network [25]. However, to our knowledge it has not yet been studied whether the necessary connectivity can emerge during reward-based learning with a biologically plausible learning rule. Can the network architecture illustrated in Fig 4, which learns to link colinear line elements, also learn to trace a curve? If yes, will learning induce a propagation of enhanced neuronal activity along the relevant curve, just as is observed in the visual cortex of monkeys? We had to make adjustments to the input layer of the network because of the different format of the input. The input layer and the linking layer now consisted of a 5 × 5 grid with 3 cells at every grid location to encode the possible colors; we used a unit coding for red, green and for luminance at each location. The association layer contained 25 neurons and the motor layer also consisted of a 5 × 5 grid, because each position could be a target for a specific stimulus (the model could select one of 25 eye movements for every stimulus). To ensure that the model would learn the grouping rule and that it could not solve the task by memorizing the specific eye-movements associated with a limited set of stimuli, we randomly generated a new stimulus on every trial. In the final version of the task, the stimulus always consisted of two equally long lines of two to five pixels each. We considered two pixels to be connected when they shared an edge. According to this rule the two curves in Fig 10 are not connected although they do touch each other. We randomly selected two of the end-points as potential eye movement targets and one other endpoint as the location of the cue. It was the model’s task to select an eye movement to the green target that was on the same curve as the red cue (Fig 9).

Researchers usually train monkeys to perform such a complex task in a number of phases in which the difficulty of the task increases gradually, a strategy that is sometimes called shaping. We used a similar strategy to shape the model, because it otherwise got stuck in a local minimum without finding a solution. We started the training procedure by presenting displays with a single square with a green eye movement target on an otherwise black background and rewarded the model for making an eye movement to this square. In the subsequent phase, we introduced the lines and the red cue, gradually increasing line length until the lines were up to five pixels long (see Suppl. A in S1 Text for details). Training was considered successful when a greedy policy (i.e. selection of the most active output unit) would lead to correct performance for 400 consecutive stimulus presentations.

We found that all 12 networks that we tested reached criterion performance with an average of 164,000 iterations (range: 136,000–189,000). This extensive training procedure would correspond to ∼ 100 training days with a monkey, with 1500 trials per day. Note, however, that we used the same learning parameters as in the contour linking task, and that we have not optimized these parameters for learning speed. Furthermore, the model learned a relatively complex version of the curve-tracing task where one of the curves was cued by a red circle and where the shapes of the two curves were much more variable than those that have been used to train monkeys. In addition, it is safe to assume that the monkeys had substantial prior experience with perceptual grouping prior to their first exposure to the curve-tracing task.

**Fig 11:**
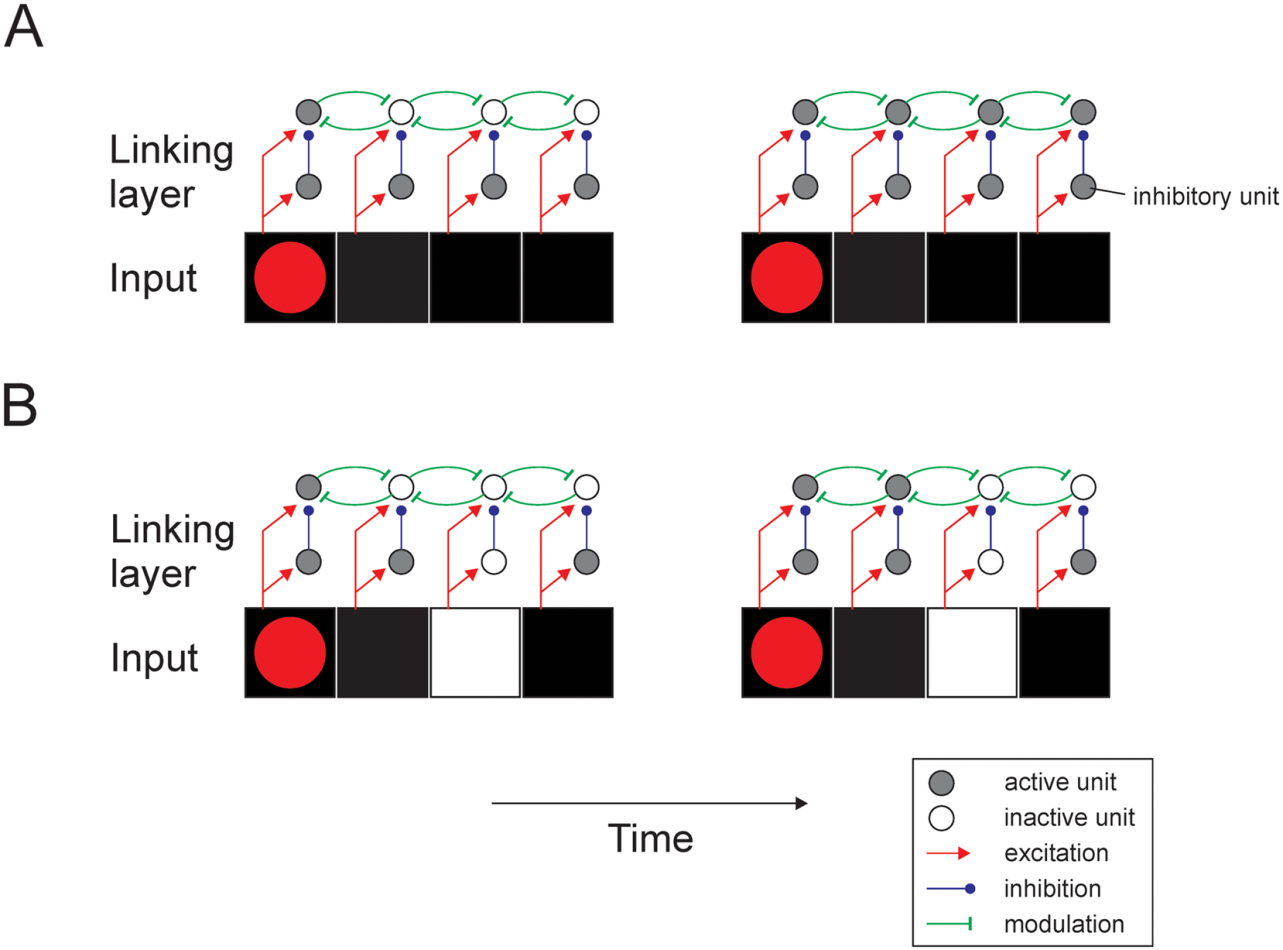
Connectivity structure established during training in the curve tracing task. **A**, Feedforward input into the linking layer causes balanced excitation and inhibition (through the inhibitory units) preventing linking layer units to become active. The unit in the linking layer tuned to red (not shown here) provides modulatory input, thereby increasing the impact of feedforward excitation to the left unit and this extra activity can spread through horizontal connections in the linking layer. **B**, If a unit in the linking layer does not receive feedforward input, horizontal modulatory influences cannot occur, thereby preventing the spread of activity across gaps in the linking layer, which is important for the detection of connectedness.

We next examined how the networks solve the task by investigating the activity that the units had acquired during the learning process. We first examined the pattern of activity in the linking layer and found that units coding for pixels of the target curve exhibited stronger activity than units coding pixels of the distractor (Fig 10). Initially, the units signaled the appearance of a pixel in their receptive field. The response enhancement occurred after a delay, because it required the spread of activity from the unit coding for the red cue in the linking layer to the unit coding for the luminance of that pixel. The enhanced activity then gradually propagated across the curve until it reached the eye movement target at the other end. The gradual spread of enhanced activity across the relevant curve is qualitatively similar to the activity in area V1 of macaque monkeys during this task, although a detailed comparison of the timing of the model (in time units) and V1 activity (in ms) is not feasible because we did not model the conduction and synaptic integration delays that determine the propagation of neuronal activity in the brain. In monkey V1, the response enhancement also did not occur during the initial visual response that was elicited by the appearance of a contour element in the neurons’ receptive fields, but after a delay of ∼ 150ms [8]. The enhanced activity then gradually spread over this curve with a speed of ∼ 50ms per receptive field until all its contour elements were labeled with enhanced neuronal activity [95]. To detect connectedness, it is important that the enhanced activity is selectively propagated along the representation of the curve and that it cannot spread to blank image locations in between curves [8]. A closer look at the connection pattern of the linking layer revealed why the network indeed only spread activity among adjacent units with a pixel in their receptive field (Fig 11). Trained networks developed balanced feedforward excitation and di-synaptic inhibition from the input layer to the linking layer (similar to neurophysiological and anatomical findings [97, 98]). Units in the linking layer were therefore not directly (or only weakly) activated by a pixel in their receptive field without concurrent modulatory input. The unit in the linking layer representing the red cue was active, however, and it had a modulatory effect that increased the impact of the excitatory input to the unit coding for luminance at the same location (see Eq (1)), causing the unit to become active. This activity then spread through the modulatory connections to the other units representing connected pixels. It did not spread to blank space, however, because the units in the linking layer did not receive excitatory input and the modulatory connections had no effect. As a result, the enhanced activity spread selectively over the target curve until it reached the other end with the green marker, thereby highlighting the correct target for an eye movement.

**Fig 12:**
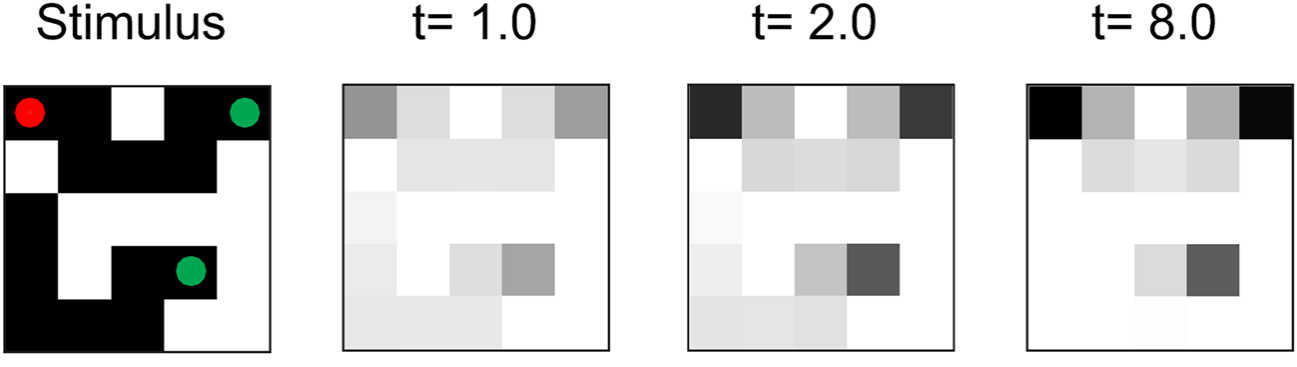
Generalization to longer line lengths. Although the model had been trained with lines with up to five pixels, it also generalized to longer line lengths. In this example stimulus, the model propagated enhanced activity over a target line of seven pixels (darker colors denote higher levels of activity).

Although we trained the models with contour lengths up to five pixels, additional tests revealed that they generalized their ability to trace a curve to longer line lengths (Fig 12). Thus, they learned a general solution to the curve-tracing task, which they could apply to new contour configurations that had not been presented previously. A trained model with a greedy policy solved about 97% of 10,000 random stimuli with contours of length 5 and about 81% with length 6. To our knowledge, RELEARNN is the first biologically realistic model that can train a neural network to solve the curve-tracing task. Interestingly, the model discovered the strategy to propagate enhanced neuronal activity from the cue over the rest of the curve, which is also used by monkeys trained on this task. The analysis of the established connectivity patterns presents a prediction for future neuroscientific studies, which can now examine the influence of learning curve-tracing and contour-linking tasks on the patterns of connections between neurons in early areas of the visual cortex.

In our standard networks, units of the linking layer were only connected to units with nearby receptive fields. We also investigated if networks with full connectivity in the linking layer learn the curve-tracing task. We found that they did and that connections between units in the linking layer with nearby receptive fields became strongest.

Finally, we tested if it is possible to train networks without horizontal connections in the linking layer to trace a curve. We found that learning did not occur once line length increased beyond three pixels. Previous studies demonstrated that the minimal number of association units that are required to detect connectedness in feedforward networks with a similar architecture increases very rapidly with line length [96, 99, 100], implying that biologically realistic implementations of algorithms for the detection of connectedness strongly benefit from recurrent connections.

## Discussion

We have presented a new biologically inspired learning rule that explains how a recurrent neural network can learn to perform contour-linking and curve-tracing tasks by adjusting the strengths of excitatory, inhibitory and modulatory connections. RELEARNN extends previous biologically realistic learning rules relying on two factors to gate Hebbian plasticity, a reward-prediction error and feedback from the response selection stage [34, 35], to recurrent neural networks. Our main result is that RELEARNN can change the attractor states of recurrent networks to improve the representation of action values. RELEARNN updates action values by a gradient descent with an accessory network for the propagation of credit assignment signals, in combination with a system that computes reward prediction errors. Jointly, these two factors ensure that the information for changing synaptic strength is available locally, within the cortical column.

We tested RELEARNN in two tasks that have been used to investigate perceptual organization in monkeys. We chose these tasks because previous work suggested that they are not solved in a feedforward manner in the brain but rely on recurrent processing, i.e. the recirculation of activity through feedforward, horizontal and feedback connections. Such processing has previously been formalized in counter stream architectures that can combine hypotheses at lower and higher hierarchical network levels through feedforward and feedback streams [66–69]. The first task was a contour linking task (Fig 1A). We found that RELEARNN was able to train a recurrent network to detect colinear patterns. Interestingly, learning enhanced the representation of line elements of the target contour in the linking layer of the network, propagating extra activity through horizontal and feedback connections. The response enhancement therefore did not occur during the initial response, but after a delay, just as has been observed for neurons in the visual cortex of monkeys trained in this task [14, 16, 101]. The model predicts that learning in this task should strengthen the lateral connections between neurons coding colinear line elements. Interestingly, RELEARNN could also train feedforward networks to detect colinear patterns, although feedforward networks do not explain the delayed influence of colinearly aligned image elements on neuronal activity in early visual cortex.

The second task was a curve-tracing task where the model had to determine the connections between adjacent pixels in order to determine the location of a target of a saccadic eye movement. The development of a learning rule that permits the detection of connectedness by a neural network is of some theoretical interest, because connectedness detection was one of the examples that Minsky and Papert [96] gave to demonstrate that the perceptual capabilities of perceptrons (feedforward networks with two layers) are limited and that some of these limitations can be alleviated by serial processing, e.g. by Turing machines. One task that relies on connectedness detection is curve-tracing (Fig 1B) [102]. Observers determine which contour elements belong to a single, connected image component, and processing in this task is indeed serial as reaction times increase linearly with the length of curve that needs to be traced [18]. Without horizontal connections, our networks did not learn the task for contours longer than three pixels, as predicted by previous studies [96, 99, 100]. Psychological research established that human observers gradually spread object-based attention across the relevant curve when they trace it [93]. At a neuronal level, the tracing of a curve is associated with the gradual spread of enhanced neuronal activity along the curve [8, 95]. It is remarkable that a neural network trained with RELEARNN developed the same strategy, gradually labeling the relevant curve with enhanced activity in the linking layer, until the enhanced response reached the saccade target so that it could be selected for an eye movement response.

The excellent correspondence between our modeling results and neurophysiological data in two different tasks suggests that RELEARNN captures properties of cortical learning. Our approach differs from previous studies addressing the connectedness problem like, for example, [100], because we proposed a biologically plausible learning rule, which permits a detailed analysis of the established connectivity structures and activity patterns that can be compared to neurophysiological findings. RELEARNN uses two factors to gate Hebbian plasticity, in accordance with previous modeling work, but it generalized the proposed learning rules to recurrent networks. The first factor is a reward-prediction error that has been central to many recent advances in biologically inspired learning [41, 81]. It is likely that the reward-prediction error is made available to neurons in cortical and subcortical structures by the release of neuromodulators, such as dopamine. Schultz and his co-workers demonstrated that the firing rate of dopamine neurons in the substantia nigra and ventral tegmental area code reward-prediction errors, i.e. the difference between the amount of reward that was anticipated by a monkey and the amount of reward that was actually received [37]. Furthermore, dopamine is known to influence synaptic plasticity so that it could fulfill a role in gating plasticity [103]. However, it is conceivable that other neuromodulators such as acetylcholine or seretonin may play equivalent roles.

The second factor used by RELEARNN is a feedback signal initiated by the selected action, which propagates through the accessory network. RELEARNN ensures that connections of the accessory network are reciprocal to those of the regular network, so that the accessory units in the columns with a large influence on the estimated value of the selected action are very active (this reciprocitity can emerge during the learning process itself [34]). The top-down influence of action selection on activity at earlier processing levels is known as an effect of selective attention in psychology and it has also been studied in neurophysiological work. Specifically, if a subject selects a stimulus for an eye or hand movement, selective attention is directed to that stimulus [104, 105], and selective attention gates learning [106, 107]. Moreover, research with the curve-tracing task revealed that action selection indeed also influences neuronal activity in the visual cortex of monkeys. In the visual cortex, a curve that is selected for a behavioral response elicits stronger neuronal activity than a curve that is not selected [108], even if the selected response is wrong (i.e. if the monkey makes an error [17, 21]). RELEARNN combines the accessory feedback effect caused by action selection with information about the reward-prediction error that is globally available through the release of neuromodulators to improve the value estimate of the selected action.

In what follows, we will first discuss the relation between RELEARNN and the Almeida-Pineda algorithm for the learning of attractor states in recurrent neural networks [82, 87]. We will then compare RELEARNN to other models for learning in recurrent neural networks and discuss its relation to previous models of perceptual grouping. RELEARNN may open a new path towards unifying theoretical and experimental research in a number of different fields: the neurophysiology and psychology of perceptual organization and the role of object-based attention therein, reinforcement learning theory and the role of feedforward and feedback connections in cortical computation.

### Comparison With the Almeida-Pineda Algorithm

RELEARNN is best understood as a biologically inspired implementation of the supervised Almeida-Pineda learning algorithm for recurrent neural networks [82, 87]. The synaptic changes of RELEARNN are proportional to those that the Almeida-Pineda learning algorithm would take to adjust the value of the selected action (Suppl. C in S1 Text). There are two potential issues that are inherited from the Almeida-Pineda algorithm. The first is related to the convergence of the regular network to a stable attractor. It is difficult to derive general conditions that can guarantee that non-symmetric networks converge to a stable attractor [87, 109]. We therefore had to rely on our simulations where we found that without strong mutual inhibition (that we omitted in the network design to reproduce the experimental findings) the network always was attracted to a stable state. In contrast, the associate network is linear and it will converge provided that the regular network is at a fixed point as was shown by [87]. The second stability issue is related to the solution that is found for the task. Although RELEARNN makes adjustments to the synaptic weights that improve the estimated value of the selected action by gradient descent, there are no guarantees that such a network will find an appropriate solution. Indeed, any gradient descent may get stuck in a local minimum in weight space where further improvements will not occur. In the curve-tracing task, for example, the learning only succeeded when we gradually increased the contour length. Without such a shaping strategy the network did not find a successful strategy to solve the task but got stuck in a local minimum.

### Comparison With Other Methods for Learning in Recurrent Neural Networks

Learning is one of the most important topics in neural networks research. Learning in recurrent neural networks (RNNs) is more complex than in feedforward networks, because a recurrent network typically has to evolve to a stationary state. This increase in complexity is offset by the advantage that RNNs can learn to compute and store intermediate computational results through their internal feedback structure. This property of RNNs is essential if the network has to remember previous inputs in a time series (time lagged recurrent networks; TLRNs, [110, 111]) but it can also be helpful in the analysis of stationary patterns, as was the case in our simulations (simultaneous recurrent networks; SRNs, [100]). In the curve-tracing task, the RNN learned to inject activity at the cue and to gradually spread this activity over the curve. It found a serial solution for a task that is difficult to solve for a feedforward network [96, 99], because connectedness is a transitive property. If pixel A is connected to pixel B and B to C, then the network should infer that A is also connected to C. The network learned to compute connectedness by serially evaluating the connectedness of adjacent pixels, first spreading the enhanced activity from pixel A to B and then onwards from B to C. This process appears to correspond well to the primitive form of serial reasoning that humans and monkeys employ to solve this task. Similar processing has been employed in other (non-biological) computational models as well [100]. Thus, in the curve-tracing example, the intermediate computational results correspond to the set of pixels that have so far been labeled with enhanced activity.

The learning of stable states of the network as implemented by RELEARNN differs from other recurrent neural network learning schemes, such as backpropagation through time (BPTT; [112]), recirculation algorithms [113–115] and reservoir computing [116, 117]. BPTT [112] was the first method for learning by recurrent neural networks. The successive network states are unfolded over time, and the learning rule is equivalent to standard backpropagation algorithm for the unfolded network. A disadvantage of BPTT is that the unfolded network requires a lot of memory (the memory footprint of a single iteration times the number of iterations) and it does not seem to be biologically plausible. Another disadvantage is that the error gradients become very small after a number of time steps, a limitation that has been alleviated by the long short term memory model (LSTM) designed for processing time-series [110, 118, 119]. RELEARNN also differs from recirculation algorithms (such as GeneRec [113]), which require symmetric connections between network units. They compare the activity of network units in two phases. The first is called “minus phase” in which the input is provided to the network until it settles in a stable state. This is followed by a “plus phase” where the activity of some of the units is clamped to a target pattern. Learning is based on the comparison between the unit activity between phases, which provides a measure for the influence of the units on the target pattern. An important difference is that RELEARNN computes action values and uses a separate accessory network in combination with a reward prediction error to determine the change in synaptic weights. Unlike the recirculation algorithms, it can therefore also train networks with non-symmetric weights.

RELEARNN also differs from reservoir computing methods, such as echo state networks [116, 117], liquid state networks [120] and backpropagation-decorrelation learning [121]. These learning schemes apply an input sequence to a randomly connected recurrent neural network, the “reservoir”, and learning takes place by changing connections between the reservoir neurons and the network’s output [122]. In RELEARNN learning also takes place within the RNN itself, so that the network can construct useful new representations of intermediate computational results.

### Comparsion With Other Reinforcement Learning Methods

Motivated by findings that midbrain dopamine neurons encode a quantitative reward prediction error [37, 123, 124], we employed temporal difference learning [41] with a SARSA style prediction error for immediate rewards. Each action is associated with an expected return value when performing this particular action in a given state. The learning algorithm tries to minimize the difference between the current estimate and the observed one. Another route of optimization has been described by policy gradient methods (such as REINFORCE) [125, 126] which directly optimize the expected reward instead of the value function. The learning rule in this case reads Δ*W*_*kl*_ = *η*(*ϱ* − *b*) ⋅ ∂ log(*ϕ*_*l*_)/∂*W*_*kl*_, where *b* denotes an arbitrary reinforcement baseline and *ϕ*_*l*_ denotes the probability of selecting action *l* (e.g. Eq (5)). Note that this equation looks similar to Eq (8) at first sight but results in an entirely different update rule. For the softmax rule Eq (5), for example, the derivative is given by

$$\begin{array}{c} \frac{\partial \log(\varphi_{l})}{\partial W_{kl}}=\frac{\partial }{\partial W_{kl}}\log(\frac{\exp(p_{l}/\tau )}{\sum_{j}\exp\left(p_{j}/\tau \right)}) \end{array}$$ (32)

$$\begin{array}{c} =\frac{\sum \exp(p_{j}/\tau )}{\exp(p_{l}/\tau )}\cdot \frac{\frac{1}{\tau }\exp\left(p_{l}/\tau \right)\frac{\partial }{\partial W_{kl}}p_{l}\sum_{j}\exp\left(p_{j}/\tau \right)-\exp\left(p_{l}/\tau \right)\sum_{j}\exp\left(p_{j}/\tau \right)\cdot \frac{1}{\tau }\cdot \frac{\partial }{\partial W_{kl}}p_{j}}{{\left(\sum_{j}\exp(p_{j}/\tau \right)}^{2}} \end{array}$$ (33)

$$\begin{array}{c} =\frac{\frac{1}{\tau }\sum_{j}\exp\left(p_{j}/\tau \right)\cdot \frac{\partial }{\partial W_{kl}}\left[p_{l}-p_{j}\right]}{\sum_{j}\exp\left(p_{j}/\tau \right)} \end{array}$$ (34)

The derivatives ∂/∂*W*_*kl*_[*p*_*l*_ − *p*_*j*_] are given by Eq (18) but it is unclear how to derive an equation like Eq (29) to avoid the calculation of the matrix inverse and the non-local sum over all units. Additionally, policy gradient has been shown not to work in all cases. A previous study [127], for example, had to add Hebbian terms in order to solve a particular task which are already part of RELEARNN (c.f. Eq (8)).

### Previous Models of Perceptual Grouping

We used RELEARNN to train networks to perform tasks that require perceptual grouping of image elements. It is therefore of interest to compare our results to previous models addressing the grouping of image elements into surfaces and boundaries (see e.g. [128], for a review). Previous models examined the contributions of horizontal connections (e.g. within area V1) to perceptual grouping by enhancing the responses elicited by colinear contour elements [129–131]. Other models aimed to explain how multiple cortical modules interact to enable the correct interpretation of a visual scene [132] or how the modification of long range horizontal connections by top-down interaction can account for such findings [22]. These previous models proposed various network structures, whereas the present study investigated how these grouping operations can be learned. We started from networks with random connectivity, and investigated learning if the only feedback from the environment is a reward upon correct task performance, and we thereby complemented previous studies addressing the role of visual experience in the tuning of cortico-cortical connections [114, 133, 134].

The present results are also relevant for a current debate about the locus of perceptual learning. A few days of experience with the contour grouping task improves the performance of monkey and human subjects [14, 135]. On the one hand, it has been suggested that these improvements are the result of a more efficient read out of sensory representations so that the sensory representations themselves can remain relatively stable [136]. On the other hand, other studies provided compelling evidence that the early sensory representations themselves can also be subject to plasticity in the adult [27, 137, 138]. RELEARNN permits the modification of connections at multiple stages. The connections from the association layer to the motor selection stage exhibit plasticity. However, synaptic changes can also occur at earlier processing levels when the synaptic modifications in higher areas do not suffice to solve the task, in accordance with previous theoretical proposals of a reversed hierarchy in perceptual learning [29, 139].

### Possible Improvements and Extensions

One limitation of the model is that learning cannot occur in networks that fail to reach a steady state. Although we did not encounter unstable states in our simulations, strong excitatory-inhibitory (E-I) interactions can lead to limit-cycles [140, 141]. Possible extensions of the model might permit learning while the system is oscillating, although this has not yet been explored by us. Another limitation is that we only considered tasks with immediate reward and did not simulate delayed reward tasks where the model has to go through several states in the environment before it can obtain the reward. It is possible to use biologically plausible learning rules with the same two factors, i.e. feedback from the response selection stage in combination with a reward-prediction error, to solve delayed reward tasks [35, 36]. These approaches increase the learning rate by incorporating information about previous activations as synaptic eligibility traces and may thereby expand the capabilities of RELEARNN. More precisely, after selecting an action the model would need to store the correlation between the activity of the regular and the accessory units in some form of synaptic tag. Upon reward delivery the neuromodulator that provides the global learning signal could interact with this tag to shape the network (c.f. [36]). In the simulations we chose a minimal model architecture that was motivated by findings of modulatory feedback interactions [43, 44, 142]. It will be of interest to systematically investigate further model variants with, for example, additional layers so that the networks can also learn to optimize the sensory features. Future studies can also address networks where feedback connections drive excitatory and/or inhibitory units at lower network levels instead of only modulating them multiplicatively [143]. In addition, future studies could incorporate more realistic measures for propagation and synaptic integration delays in the networks to enable a more precise comparison between activity propagation in the networks and the spread of neuronal activity in the visual cortex as well as the pattern of reaction times of human observers who carry out the curve-tracing task.

### Conclusion

We have presented a new learning rule that explains how recurrent neural networks can learn to group image elements during contour linking and curve-tracing. The correspondence between the simulation results and the neurophysiological data obtained in monkeys trained on these tasks suggests that the proposed learning algorithm captures the influence of learning on interactions between neurons in the visual cortex. RELEARNN explains how a neural network can learn incremental, transitive grouping operations by the spread of enhanced neuronal activity by trial and error and how it can exploit these grouping results to guide behavior. We anticipate that future studies may build on these results in the search for even more powerful methods to impose structure on incoming sensory data.

## Supporting Information

S1 Text Simulation parameters and details of the derivation. (PDF)
