# Supplementary material for: Reinforcement Learning of Linking and Tracing Contours in Recurrent Neural Networks
Source: PLoS Comput Biol. 2015 Oct 23;11(10):e1004489. doi: 10.1371/journal.pcbi.1004489 (PMC4619762; doi:10.1371/journal.pcbi.1004489)
Supplement: S1 Text — (PDF) [file pcbi.1004489.s001.pdf]

Text S1

Simulation parameters and detailed derivations for reinforcement learning of linking and tracing contours in recurrent neural networks

A Simulation Procedures: Parameters and Test Settings

We used RELEARNN to train neural networks on two different tasks. The first task was to link visual contours and the second task was curve tracing. Parameters in both experiments have been set to $\alpha = \beta = \zeta = 1$, $\gamma = 4$, $a = 0.001$ (c.f. eqn. (1)) and the reward was set to $\varrho = 0$ and $\varrho = 0.4$ in case of error and success, respectively. We performed simulations with the one step Euler-method with $\Delta t = 0.04$ iterating until convergence ($\varepsilon = 0.0001$). Weights were bounded by $\theta^{high} = 4$ by adapting the weights according to $W \mapsto W + \Delta W \cdot (\theta^{high} - W)$ to accomodate the fact that neural connectivity cannot be arbitrarily large. However, in practice the maximal value of any weight in the networks was ≈ 2. Excitatory and inhibitory connections to the linking layer were initialized with values randomly chosen from the interval $[0.98, 1.02]$. Connections to (excitatory) and from (modulatory) the association layer were initialized with small weights randomly chosen from the interval $[0.001, 0.02]$. To speed up learning in the tracing task each motor unit was "paired" with an association unit from which it received strong excitation randomly initialized with values uniformly drawn from $[0.95, 1.05]$. Training simulations conducted without this high initialization weights were also successful but took longer. The lateral modulatory connections were initialized with values randomly drawn from $[0.001, 0.05]$ (to speed up learning in the tracing task, we initialized them with $[1.45, 1.55]$). The learning rate η was set to 0.65 and the parameter τ of the softmax rule was set to 0.004.

A.1 Learning to Link Contours

We presented stimuli within two apertures with randomly oriented bars to the input layer of the network on a 9×9 grid with units selective to four orientations. The activity r of a neuron tuned to orientation Θ elicited by a bar with orientation ϑ equalled $r = \exp(\kappa \cdot \cos(2 \cdot (\vartheta - \Theta)) - \kappa) - |\chi|$, with $\kappa = .9$ and a Gaussian noise χ with standard deviation 0.65 to account for the jitter used in the experiments of [14]. There were a total of two motor units that signaled the hemisfield with the target contour and four association layer units. We did not include interhemispheric connections between units in the linking layer that represented input patterns on either side of the fixation point.

A.2 Curve-Tracing Task

In the curve-tracing task, the visual input was presented on a 5×5 grid of neurons selective for luminance and for the colors red and green. To decrease the computational costs, we did not model orientation selective responses as they do not encode information that was relevant for the present task. The linking layer also consisted of $5 \times 5 \times 3$ neurons, receiving input from the units selective for luminance, red and green. The association layer contained 25 neurons and the motor layer consisted of a 5×5 grid of possible saccade targets, because every pixel in the input layer could be selected for a saccade.

We started with a pre-training phase where we trained the model to make saccades to green dots, thereby emulating the shaping strategy used to train monkeys in comparable tasks. In the pre-training phase, we set the activity of the neurons selective for green and luminance to 0.7 and discontinued pre-training when a greedy decision policy (i.e. selection of the maximally active output neuron) would have succeeded for 400 times in a row.

In the curve-tracing task, we generated two lines of equal length. The activity of luminance input neurons was 0.1, the activity of the neurons coding red at the cued location was 0.7 and the activity

of neurons coding the green saccade targets was 0.7. We started with curves with a length of 2 and we gradually increased line length (choosing random configurations of adjacent pixels for the target and distractor curve) up to a length of 5 whenever a greedy-policy would have been successful for 400 stimulus presentations in a row.

B Taylor-Expansion

Here, we derive the Taylor expansion of the full dynamics given in eqn. (3). We define the right-hand side of eqn. (3) as

$$f(\boldsymbol{p}) = -\mathrm{diag}(\alpha)\boldsymbol{p} + \mathrm{diag}(\beta - \boldsymbol{p}) \cdot \mathrm{diag}(\underbrace{(\boldsymbol{W}^{ex})^T g(\boldsymbol{p}) + (\boldsymbol{W}^{I})^T \boldsymbol{I}^{inp}}_{\boldsymbol{I}^{ex}}) \cdot (1 + \gamma \underbrace{(\boldsymbol{W}^{mod})^T g(\boldsymbol{p})}_{\boldsymbol{I}^{mod}}) - \mathrm{diag}(\zeta + \boldsymbol{p}) \cdot \underbrace{(\boldsymbol{W}^{inh})^T g(\boldsymbol{p})}_{\boldsymbol{I}^{inh}} \,. \quad (35)$$

This is, for a single element p_i

$$f(p_i) = -\alpha p_i + (\beta - p_i) \cdot \left(\sum_{j=1}^{N} W_{ji}^{ex} g(p_j) + \sum_{j=1}^{N} W_{ji}^{I} I_j^{inp} \right) \cdot \left(1 + \gamma \cdot \sum_{j=1}^{N} W_{ji}^{mod} g(p_j) \right) - (\zeta + p_i) \cdot \sum_{j=1}^{N} W_{ji}^{inh} g(p_j) \,. \quad (36)$$

To determine the Taylor expansion of f up to first order, we only need to determine $d/d\boldsymbol{p} f$ since $f(\boldsymbol{p}^{\infty}) = 0$. Derivation with respect to $\boldsymbol{p}$ according to matrix calculus results in

$$\frac{d}{d\boldsymbol{p}} f(\boldsymbol{p}) = -\mathrm{diag}(\alpha) - \mathrm{diag}\left[\boldsymbol{I}^{ex} \cdot (1 + \gamma \boldsymbol{I}^{mod})\right] + \mathrm{diag}\left[(\beta - \boldsymbol{p})(1 + \gamma \boldsymbol{I}^{mod})\right] (\boldsymbol{W}^{ex})^T \boldsymbol{G} - \gamma \cdot \mathrm{diag}\left[(\beta - \boldsymbol{p}) \cdot \boldsymbol{I}^{ex}\right] (\boldsymbol{W}^{mod})^T \boldsymbol{G} - \mathrm{diag}(\boldsymbol{I}^{inh}) - \mathrm{diag}(\zeta + \boldsymbol{p})(\boldsymbol{W}^{inh})^T \boldsymbol{G} =: \boldsymbol{\mathcal{L}}^T \,, \quad (37)$$

with

$$\boldsymbol{G} = \mathrm{diag}\left[\frac{d}{d\boldsymbol{p}} g(\boldsymbol{p})\right] . \quad (38)$$

C Connection to Almeida-Pineda: Derivation of Supervised Learning in Recurrent Neural Networks (LEARNN)

In this section, we apply the Almeida-Pineda algorithm [82,87] for the neuron type described in the Models section. We derive a supervised learning algorithm for recurrent neural networks which we call LEARNN. We will then adapt the teaching signal to learn from experience and demonstrate that LEARNN in this reinforcement setting is identical to the RELEARNN algorithm using gated Hebbian plasticity.

As in the Almeida-Pineda method, we assume that the system eqn. (3) reaches a stable steady state $\boldsymbol{p}^{\infty}$. Although little is known about the stability of the network system eqn. (3) for arbitrary weight matrices our numerical simulations confirmed the observations of [82] and [109] of similar systems that

the dynamics tend to converge to a stable state. Furthermore, the boundedness of the activities in system eqn. (3) guarantees that the system stays within these bounds. The learning mechanism minimizes an error function that reflects deviation from the target values τ_j

$$E(x) = \frac{1}{2}\sum_{i=1}^{N} J_i^2\,, \quad \text{where} \qquad J_i = \begin{cases} \tau_i - p_i^\infty\,, & i \in \mathcal{O}\,, \\ 0\,, & \text{otherwise.} \end{cases} \tag{39}$$

Weight changes are calculated by gradient-descent with learning rate η (η must be small so that $\boldsymbol{p}$ is essentially at steady state, i.e. $\boldsymbol{p} \approx \boldsymbol{p}^\infty$)

$$\Delta W_{kl}^{(\cdot)} = -\eta \frac{\partial E}{\partial W_{kl}^{(\cdot)}} = \eta \sum_{i=1}^{N} J_i \frac{\partial p_i^\infty}{\partial W_{kl}^{(\cdot)}}\,. \tag{40}$$

In the next step, we derive the values of $\partial p_i^\infty / \partial W_{kl}^{(\cdot)}$, i.e. how a small change of weight W_{kl} influences the activity of neuron p_i at equilibrium. Applying matrix calculus (e.g. [89]; c.f. appendices C.1 to C.4), we calculate the weight changes such that

$$\frac{\partial p_i^\infty}{\partial W_{kl}^{Inp}} = -(\mathcal{L}^{-1})_{li} \cdot (\beta - p_l^\infty) \cdot (1 + \gamma (I_\infty^{mod})_l) \cdot p_k^{inp}\,, \tag{41}$$

$$\frac{\partial p_i^\infty}{\partial W_{kl}^{ex}} = -(\mathcal{L}^{-1})_{li} \cdot (\beta - p_l^\infty) \cdot (1 + \gamma (I_\infty^{mod})_l) \cdot g(p_k^\infty)\,, \tag{42}$$

$$\frac{\partial p_i^\infty}{\partial W_{kl}^{inh}} = (\mathcal{L}^{-1})_{li} \cdot (\zeta + p_l^\infty) \cdot g(p_k^\infty)\,, \tag{43}$$

$$\frac{\partial p_i^\infty}{\partial W_{kl}^{mod}} = -\gamma \cdot (\mathcal{L}^{-1})_{li} \cdot (\beta - p_l^\infty) \cdot (I_\infty^{ex})_l \cdot g(p_k^\infty)\,, \tag{44}$$

where[1]

$$\begin{aligned}\boldsymbol{\mathcal{L}}^T = &-\text{diag}[\alpha] - \text{diag}\left[\boldsymbol{I}_\infty^{ex} \cdot \left(1 + \gamma \boldsymbol{I}_\infty^{mod}\right)\right] - \text{diag}\left[\boldsymbol{I}_\infty^{inh}\right] + \\ &\text{diag}\left[(\beta - \boldsymbol{p}^\infty) \cdot \left(1 + \gamma \boldsymbol{I}_\infty^{mod}\right)\right] (\boldsymbol{W}^{ex})^T \boldsymbol{G} - \text{diag}\left[\zeta + \boldsymbol{p}^\infty\right] \cdot \left(\boldsymbol{W}^{inh}\right)^T \boldsymbol{G} + \\ &\gamma \cdot \text{diag}\left[(\beta - \boldsymbol{p}^\infty) \cdot \boldsymbol{I}_\infty^{ex}\right] \cdot \left(\boldsymbol{W}^{mod}\right)^T \boldsymbol{G}\end{aligned} \tag{45}$$

with

$$\boldsymbol{G} = \text{diag}\left[\frac{d}{d\boldsymbol{p}} g(\boldsymbol{p})\right]\,. \tag{46}$$

See sections C.1 to C.4 for a detailed derivation of eqns. (41) to (45). Here, $\text{diag}[\alpha] \in \mathbb{R}^{N\times N}$, $\alpha \in \mathbb{R}$ denotes a diagonal matrix, where each element a_{ii} has the value α and $\text{diag}[\boldsymbol{p}] \in \mathbb{R}^{N\times N}$, $\boldsymbol{p} \in \mathbb{R}^N$ is a diagonal matrix with $a_{ii} = p_i$. Note that eqns. (41) to (44) require a matrix inversion. Instead of calculating the matrix inverse, we introduce an *associated dynamical system* utilizing a population of neurons n_j^z, $j = 1 \ldots N$ with activities z_j

$$\dot{\boldsymbol{z}} = \boldsymbol{\mathcal{L}} \boldsymbol{z} + \boldsymbol{J} \tag{47}$$

[1]Note that this $\boldsymbol{L}$ is identical to the $\boldsymbol{L}$ used in the derivation of the gated Hebbian plasticity but that it is derived by calculating the derivative of $\boldsymbol{p}^\infty$ with respect to the weights.

where $\boldsymbol{J}$ denotes the deviation of activations from the target values eqn. (39) and $\boldsymbol{L}$ is defined as in eqn. (45). At steady state the following algebraic condition holds:

$$z_l^\infty = -(\boldsymbol{\mathcal{L}}^{-1}\boldsymbol{J})_l = -\sum_{i=1}^{N}(L^{-1})_{li}J_i\,. \tag{48}$$

This steady state equation can be utilized to avoid computing the matrix inverse in eqns. (41) to (44). The resulting learning rules can now be derived as local mechanisms by combining eqns. (41) to (44), eqn. (40) and eqn. (48):

$$\begin{aligned}
\Delta W_{kl}^{Inp} &= \eta \cdot z_l^\infty \cdot (\beta - p_l^\infty) \cdot \big(1 + \gamma (I_\infty^{mod})_l\big) \cdot p_k^{inp}\,, &(49)\\
\Delta W_{kl}^{ex} &= \eta \cdot z_l^\infty \cdot (\beta - p_l^\infty) \cdot \big(1 + \gamma (I_\infty^{mod})_l\big) \cdot g(p_k^\infty)\,, &(50)\\
\Delta W_{kl}^{inh} &= -\eta \cdot z_l^\infty \cdot (\zeta + p_l^\infty) \cdot g(p_k^\infty)\,, &(51)\\
\Delta W_{kl}^{mod} &= \eta \cdot \gamma \cdot z_l^\infty \cdot (\beta - p_l^\infty) \cdot (I_\infty^{ex})_l \cdot g(p_k^\infty)\,, &(52)
\end{aligned}$$

where z_l^∞ is the steady state eqn. (48) of the associated network denoted in eqn. (47).

Equations (3), (47) and (49) to (52) completely specify the dynamics of LEARNN provided that (3) and (47) are convergent. It is known that convergence of a system of neurons with activation dynamics as defined in eqn. (3) is a sufficient condition for the convergence of (47) [87,88]. To ensure boundedness, positivity of the elements of the weight matrices must be enforced in each step, i.e. weights are rectified according to $W_{ij}^{(\cdot)} = \max\{0, W_{ij}^{(\cdot)}\}$.

Now LEARNN can be easily extended to reinforcement learning. Like described in the Models section, the network acts as a reinforcement learning agent. The state is presented as input to the network and the output units engage in a competition that selects one of a number of actions. We employ a SARSA-style return as in eqn. (7) and define the teaching signal of LEARNN as

$$J_i = \begin{cases} \rho - p_a(s)\,, & i = a\,,\\ 0\,, & i \neq a\,. \end{cases} \tag{53}$$

We can now compare eqn. (50) (LEARNN; recurrent gradient descent) and eqn. (8). When we factor out the SARSA-term for immediate rewards $\delta := \rho - p_a(s)$ in eqn. (48), we note that LEARNN in the reinforcement setting (recurrent gradient descent) is identical to RELEARNN (gated Hebbian plasticity).

C.1 Derivative with Respect to the Input Weights

This section demonstrates how eqn. (41) is derived. We start with the derivative $\frac{\partial}{\partial W_{kl}^{Inp}} p^\infty$: At equilibrium, $\frac{d}{dt}\boldsymbol{p} = 0$, so that eqn. (3) changes to

$$\begin{aligned}
\mathbf{0} = -\mathrm{diag}(\alpha)\boldsymbol{p}^\infty + &\\
\mathrm{diag}(\beta - \boldsymbol{p}^\infty) \cdot \mathrm{diag}(\underbrace{(\boldsymbol{W}^{ex})^T g(\boldsymbol{p}^\infty) + (\boldsymbol{W}^{I})^T \boldsymbol{I}^{inp}}_{\boldsymbol{I}_\infty^{ex}}) \cdot (1 + \gamma\, \underbrace{(\boldsymbol{W}^{mod})^T g(\boldsymbol{p}^\infty)}_{\boldsymbol{I}_\infty^{mod}}) - &\\
\mathrm{diag}(\zeta + \boldsymbol{p}^\infty) \cdot \underbrace{(\boldsymbol{W}^{inh})^T g(\boldsymbol{p}^\infty)}_{\boldsymbol{I}_\infty^{inh}}\,. &\quad (54)
\end{aligned}$$

For simplicity, we omit the ∞ and just write $\boldsymbol{p}, \boldsymbol{I}^{ex}$ instead of $\boldsymbol{p}^{\infty}, \boldsymbol{I}^{ex}_{\infty}$. Derivation with respect to W^{Inp}_{kl} according to matrix calculus results in

$$\begin{aligned}\mathbf{0} = &-\mathrm{diag}(\alpha)\frac{\partial}{\partial W^{Inp}_{kl}}\boldsymbol{p} - \mathrm{diag}(\boldsymbol{I}^{ex})\mathrm{diag}\left(1+\gamma\boldsymbol{I}^{mod}\right)\frac{\partial}{\partial W^{Inp}_{kl}}\boldsymbol{p} \\ &+ \mathrm{diag}(\beta-\boldsymbol{p})\mathrm{diag}\left(1+\gamma\boldsymbol{I}^{mod}\right)\left((\boldsymbol{W}^{ex})^T\boldsymbol{G}\frac{\partial}{\partial W^{Inp}_{kl}}\boldsymbol{p} + \underbrace{\boldsymbol{E}_{lk}\boldsymbol{p}^{inp}}_{=\boldsymbol{e}_l\boldsymbol{p}^{inp}_k}\right) \\ &+ \gamma\cdot\mathrm{diag}(\beta-\boldsymbol{p})\mathrm{diag}(\boldsymbol{I}^{ex})\left(\boldsymbol{W}^{mod}\right)^T\boldsymbol{G}\frac{\partial}{\partial W^{Inp}_{kl}}\boldsymbol{p} \\ &- \mathrm{diag}(\boldsymbol{I}^{inh})\frac{\partial}{\partial W^{Inp}_{kl}}\boldsymbol{p} - \mathrm{diag}(\zeta+\boldsymbol{p})\left(\boldsymbol{W}^{inh}\right)^T\boldsymbol{G}\frac{\partial}{\partial W^{Inp}_{kl}}\boldsymbol{p}\,,\end{aligned} \quad (55)$$

where $\boldsymbol{E}_{lk} \in \mathbb{R}^{N\times N}$ is a matrix with zeros except for $(E_{lk})_{lk} = 1$, $\boldsymbol{e}_l \in \mathbb{R}^N$ is a vector with zeros except for $(e_l)_l = 1$ and $\boldsymbol{G}$ as in eqn. (46). We can now gather all factors of $\frac{\partial}{\partial W^{Inp}_{kl}}\boldsymbol{p}$ and take them to the left-side.

$$\begin{aligned}&\Big\{\mathrm{diag}(\alpha) + \mathrm{diag}\left[\boldsymbol{I}^{ex}\cdot(1+\gamma\boldsymbol{I}^{mod})\right] - \mathrm{diag}\left[(\beta-\boldsymbol{p})(1+\gamma\boldsymbol{I}^{mod})\right](\boldsymbol{W}^{ex})^T\boldsymbol{G} - \\ &\quad\gamma\cdot\mathrm{diag}\left[(\beta-\boldsymbol{p})\cdot\boldsymbol{I}^{ex}\right]\left(\boldsymbol{W}^{mod}\right)^T\boldsymbol{G} + \\ &\quad\mathrm{diag}(\boldsymbol{I}^{inh}) + \mathrm{diag}(\zeta+\boldsymbol{p})\left(\boldsymbol{W}^{inh}\right)^T\boldsymbol{G}\Big\}\frac{\partial}{\partial W^{Inp}_{kl}}\boldsymbol{p} = \\ &\quad\mathrm{diag}(\beta-\boldsymbol{p})\mathrm{diag}\left(1+\gamma\boldsymbol{I}^{mod}\right)\boldsymbol{e}_l\cdot p^{inp}_k\,.\end{aligned} \quad (56)$$

When we define the term in curled brackets as $-\boldsymbol{\mathcal{L}}^T$, we obtain eqn. (45). Multiplication with $-\boldsymbol{\mathcal{L}}^{T^{-1}}$ results in eqn. (41).

C.2 Derivative with Respect to the Excitatory Weights

This section demonstrates how eqn. (42) is derived. We start with the derivative $\frac{\partial}{\partial W^{ex}_{kl}}\boldsymbol{p}^{\infty}$: At equilibrium, $\frac{d}{dt}\boldsymbol{p} = 0$, so that eqn. (3) changes to eqn. (54) (repeated for reader's convenience):

$$\begin{aligned}\mathbf{0} = &-\mathrm{diag}(\alpha)\boldsymbol{p}^{\infty} + \\ &\mathrm{diag}(\beta-\boldsymbol{p}^{\infty})\cdot\mathrm{diag}(\underbrace{(\boldsymbol{W}^{ex})^T g(\boldsymbol{p}^{\infty}) + \left(\boldsymbol{W}^{I}\right)^T\boldsymbol{I}^{inp}}_{\boldsymbol{I}^{ex}_{\infty}})\cdot(1+\gamma\underbrace{\left(\boldsymbol{W}^{mod}\right)^T g(\boldsymbol{p}^{\infty})}_{\boldsymbol{I}^{mod}_{\infty}}) - \\ &\mathrm{diag}(\zeta+\boldsymbol{p}^{\infty})\cdot\underbrace{\left(\boldsymbol{W}^{inh}\right)^T g(\boldsymbol{p}^{\infty})}_{\boldsymbol{I}^{inh}_{\infty}}\,.\end{aligned}$$

For simplicity, we omit the ∞ and just write $\boldsymbol{p}, \boldsymbol{I}^{ex}$ instead of $\boldsymbol{p}^{\infty}, \boldsymbol{I}^{ex}_{\infty}$. Derivation with respect to W^{ex}_{kl} according to matrix calculus results in

$$\begin{aligned}\mathbf{0} = &-\mathrm{diag}(\alpha)\frac{\partial}{\partial W^{ex}_{kl}}\boldsymbol{p} - \mathrm{diag}(\boldsymbol{I}^{ex})\mathrm{diag}\left(1+\gamma\boldsymbol{I}^{mod}\right)\frac{\partial}{\partial W^{ex}_{kl}}\boldsymbol{p}\\ &+ \mathrm{diag}(\beta-\boldsymbol{p})\mathrm{diag}\left(1+\gamma\boldsymbol{I}^{mod}\right)\Big(\underbrace{\boldsymbol{E}_{lk}g(\boldsymbol{p})}_{\boldsymbol{e}_l\boldsymbol{g}(p_k)}+(\boldsymbol{W}^{ex})^T\boldsymbol{G}\frac{\partial}{\partial W^{ex}_{kl}}\boldsymbol{p}\Big)\\ &+\gamma\cdot\mathrm{diag}(\beta-\boldsymbol{p})\mathrm{diag}(\boldsymbol{I}^{ex})\left(\boldsymbol{W}^{mod}\right)^T\boldsymbol{G}\frac{\partial}{\partial W^{ex}_{kl}}\boldsymbol{p}\\ &-\mathrm{diag}(\boldsymbol{I}^{inh})\frac{\partial}{\partial W^{ex}_{kl}}\boldsymbol{p} - \mathrm{diag}(\zeta+\boldsymbol{p})\left(\boldsymbol{W}^{inh}\right)^T\boldsymbol{G}\frac{\partial}{\partial W^{ex}_{kl}}\boldsymbol{p}\,,\end{aligned} \tag{57}$$

where $\boldsymbol{E}_{lk}\in\mathbb{R}^{N\times N}$ is a matrix with zeros except for $(E_{lk})_{lk}=1$, $\boldsymbol{e}_l\in\mathbb{R}^N$ is a vector with zeros except for $(e_l)_l=1$ and $\boldsymbol{G}$ as in eqn. (46). We can now gather all factors of $\frac{\partial}{\partial W^{ex}_{kl}}\boldsymbol{p}$ and take them to the left-side.

$$\begin{aligned}&\Big\{\mathrm{diag}(\alpha)+\mathrm{diag}\left[\boldsymbol{I}^{ex}\cdot(1+\gamma\boldsymbol{I}^{mod})\right]-\mathrm{diag}\left[(\beta-\boldsymbol{p})(1+\gamma\boldsymbol{I}^{mod})\right](\boldsymbol{W}^{ex})^T\boldsymbol{G}-\\ &\quad\gamma\cdot\mathrm{diag}\left[(\beta-\boldsymbol{p})\cdot\boldsymbol{I}^{ex}\right]\left(\boldsymbol{W}^{mod}\right)^T\boldsymbol{G}+\\ &\quad\mathrm{diag}(\boldsymbol{I}^{inh})+\mathrm{diag}(\zeta+\boldsymbol{p})\left(\boldsymbol{W}^{inh}\right)^T\boldsymbol{G}\Big\}\frac{\partial}{\partial W^{ex}_{kl}}\boldsymbol{p}=\\ &\quad\mathrm{diag}(\beta-\boldsymbol{p})\mathrm{diag}\left(1+\gamma\boldsymbol{I}^{mod}\right)\boldsymbol{e}_l\cdot g(p_k)\,.\end{aligned} \tag{58}$$

When we define the term in curled brackets as $-\boldsymbol{\mathcal{L}}^T$, we obtain eqn. (45). Multiplication with $-\boldsymbol{\mathcal{L}}^{T^{-1}}$ results in eqn. (42).

C.3 Derivative with Respect to the Inhibitory Weights

This section demonstrates how eqn. (43) is derived. We start with the derivative $\frac{\partial}{\partial W^{inh}_{kl}}\boldsymbol{p}^{\infty}$: At equilibrium, $\frac{d}{dt}\boldsymbol{p}=0$, so that eqn. (3) changes to eqn. (54) (repeated for reader's convenience):

$$\begin{aligned}\mathbf{0} = &-\mathrm{diag}(\alpha)\boldsymbol{p}^{\infty}+\\ &\mathrm{diag}(\beta-\boldsymbol{p}^{\infty})\cdot\mathrm{diag}(\underbrace{(\boldsymbol{W}^{ex})^T g(\boldsymbol{p}^{\infty})+\left(\boldsymbol{W}^{I}\right)^T\boldsymbol{I}^{inp}}_{\boldsymbol{I}^{ex}_{\infty}})\cdot(1+\gamma\underbrace{\left(\boldsymbol{W}^{mod}\right)^T g(\boldsymbol{p}^{\infty})}_{\boldsymbol{I}^{mod}_{\infty}})-\\ &\mathrm{diag}(\zeta+\boldsymbol{p}^{\infty})\cdot\underbrace{\left(\boldsymbol{W}^{inh}\right)^T g(\boldsymbol{p}^{\infty})}_{\boldsymbol{I}^{inh}_{\infty}}\,.\end{aligned}$$

For simplicity, we omit the ∞ and just write $\boldsymbol{p}, \boldsymbol{I}^{ex}$ instead of $\boldsymbol{p}^{\infty}, \boldsymbol{I}^{ex}_{\infty}$. Derivation with respect to W^{inh}_{kl} according to matrix calculus results in

$$\begin{aligned}\mathbf{0} = &-\text{diag}(\alpha)\frac{\partial}{\partial W^{inh}_{kl}}\boldsymbol{p} - \text{diag}(\boldsymbol{I}^{ex})\text{diag}\left(1+\gamma\boldsymbol{I}^{mod}\right)\frac{\partial}{\partial W^{inh}_{kl}}\boldsymbol{p}\\ &+ \text{diag}(\beta-\boldsymbol{p})\text{diag}\left(1+\gamma\boldsymbol{I}^{mod}\right)(\boldsymbol{W}^{ex})^T\boldsymbol{G}\frac{\partial}{\partial W^{inh}_{kl}}\boldsymbol{p}\\ &+ \gamma\cdot\text{diag}(\beta-\boldsymbol{p})\text{diag}(\boldsymbol{I}^{ex})(\boldsymbol{W}^{mod})^T\boldsymbol{G}\frac{\partial}{\partial W^{inh}_{kl}}\boldsymbol{p}\\ &- \text{diag}(\boldsymbol{I}^{inh})\frac{\partial}{\partial W^{inh}_{kl}}\boldsymbol{p} - \text{diag}(\zeta+\boldsymbol{p})\Big((\boldsymbol{W}^{inh})^T\boldsymbol{G}\frac{\partial}{\partial W^{inh}_{kl}}\boldsymbol{p} + \underbrace{\boldsymbol{E}_{lk}g(\boldsymbol{r})}_{\boldsymbol{e}_l g(p_k)}\Big)\,, \end{aligned} \tag{59}$$

where $\boldsymbol{E}_{lk}\in\mathbb{R}^{N\times N}$ is a matrix with zeros except for $(E_{lk})_{lk}=1$, $\boldsymbol{e}_l\in\mathbb{R}^N$ is a vector with zeros except for $(e_l)_l = 1$ and $\boldsymbol{G}$ as in eqn. (46). We can now gather all factors of $\frac{\partial}{\partial W^{inh}_{kl}}\boldsymbol{p}$ and take them to the left-side.

$$\begin{aligned}&\Big\{\text{diag}(\alpha) + \text{diag}\left[\boldsymbol{I}^{ex}\cdot(1+\gamma\boldsymbol{I}^{mod})\right] - \text{diag}\left[(\beta-\boldsymbol{p})(1+\gamma\boldsymbol{I}^{mod})\right](\boldsymbol{W}^{ex})^T\boldsymbol{G} -\\ &\gamma\cdot\text{diag}\left[(\beta-\boldsymbol{p})\cdot\boldsymbol{I}^{ex}\right](\boldsymbol{W}^{mod})^T\boldsymbol{G} +\\ &\text{diag}(\boldsymbol{I}^{inh}) + \text{diag}(\zeta+\boldsymbol{p})(\boldsymbol{W}^{inh})^T\boldsymbol{G}\Big\}\frac{\partial}{\partial W^{inh}_{kl}}\boldsymbol{p} =\\ &-\text{diag}(\zeta+\boldsymbol{p})\boldsymbol{e}_l\cdot g(p_k)\,. \end{aligned} \tag{60}$$

When we define the term in curled brackets as $-\boldsymbol{\mathcal{L}}^T$, we obtain eqn. (45). Multiplication with $-\boldsymbol{\mathcal{L}}^{T^{-1}}$ results in eqn. (43).

C.4 Derivative with Respect to the Modulatory Weights

This section demonstrates how eqn. (44) is derived. We start with the derivative $\frac{\partial}{\partial W^{mod}_{kl}}\boldsymbol{p}^{\infty}$: At equilibrium, $\frac{d}{dt}\boldsymbol{p}=0$, so that eqn. (3) changes to eqn. (54) (repeated for reader's convenience):

$$\begin{aligned}\mathbf{0} = &-\text{diag}(\alpha)\boldsymbol{p}^{\infty} +\\ &\text{diag}(\beta-\boldsymbol{p}^{\infty})\cdot\text{diag}(\underbrace{(\boldsymbol{W}^{ex})^T g(\boldsymbol{p}^{\infty}) + (\boldsymbol{W}^{I})^T\boldsymbol{I}^{inp}}_{\boldsymbol{I}^{ex}_{\infty}})\cdot(1+\gamma\underbrace{(\boldsymbol{W}^{mod})^T g(\boldsymbol{p}^{\infty})}_{\boldsymbol{I}^{mod}_{\infty}}) -\\ &\text{diag}(\zeta+\boldsymbol{p}^{\infty})\cdot\underbrace{(\boldsymbol{W}^{inh})^T g(\boldsymbol{p}^{\infty})}_{\boldsymbol{I}^{inh}_{\infty}}\,. \end{aligned}$$

For simplicity, we omit the ∞ and just write $\boldsymbol{p}, \boldsymbol{I}^{ex}$ instead of $\boldsymbol{p}^{\infty}, \boldsymbol{I}^{ex}_{\infty}$. Derivation with respect to W^{mod}_{kl} according to matrix calculus results in

$$\begin{aligned}\boldsymbol{0} = -\mathrm{diag}(\alpha)\frac{\partial}{\partial W^{mod}_{kl}}\boldsymbol{p} - \mathrm{diag}(\boldsymbol{I}^{ex})\mathrm{diag}\left(1+\gamma\boldsymbol{I}^{mod}\right)\frac{\partial}{\partial W^{mod}_{kl}}\boldsymbol{p} \\ + \mathrm{diag}(\beta-\boldsymbol{p})\mathrm{diag}\left(1+\gamma\boldsymbol{I}^{mod}\right)(\boldsymbol{W}^{ex})^T\boldsymbol{G}\frac{\partial}{\partial W^{mod}_{kl}}\boldsymbol{p} \\ + \gamma\cdot\mathrm{diag}(\beta-\boldsymbol{p})\mathrm{diag}(\boldsymbol{I}^{ex})\Big(\underbrace{\boldsymbol{E}_{lk}g(\boldsymbol{p})}_{=\boldsymbol{e}_l g(p_k)} + (\boldsymbol{W}^{mod})^T\boldsymbol{G}\frac{\partial}{\partial W^{mod}_{kl}}\boldsymbol{p}\Big) \\ - \mathrm{diag}(\boldsymbol{I}^{inh})\frac{\partial}{\partial W^{mod}_{kl}}\boldsymbol{p} - \mathrm{diag}(\zeta+\boldsymbol{p})(\boldsymbol{W}^{inh})^T\boldsymbol{G}\frac{\partial}{\partial W^{mod}_{kl}}\boldsymbol{p}\,, \quad (61)\end{aligned}$$

where $\boldsymbol{E}_{lk} \in \mathbb{R}^{N\times N}$ is a matrix with zeros except for $(E_{lk})_{lk} = 1$, $\boldsymbol{e}_l \in \mathbb{R}^N$ is a vector with zeros except for $(e_l)_l = 1$ and $\boldsymbol{G}$ as in eqn. (46). We can now gather all factors of $\frac{\partial}{\partial W^{mod}_{kl}}\boldsymbol{p}$ and take them to the left-side.

$$\begin{aligned}\Big\{\mathrm{diag}(\alpha) + \mathrm{diag}\left[\boldsymbol{I}^{ex}\cdot(1+\gamma\boldsymbol{I}^{mod})\right] - \mathrm{diag}\left[(\beta-\boldsymbol{p})(1+\gamma\boldsymbol{I}^{mod})\right](\boldsymbol{W}^{ex})^T\boldsymbol{G} - \\ \gamma\cdot\mathrm{diag}\left[(\beta-\boldsymbol{p})\cdot\boldsymbol{I}^{ex}\right](\boldsymbol{W}^{mod})^T\boldsymbol{G} + \\ \mathrm{diag}(\boldsymbol{I}^{inh}) + \mathrm{diag}(\zeta+\boldsymbol{p})(\boldsymbol{W}^{inh})^T\boldsymbol{G}\Big\}\frac{\partial}{\partial W^{mod}_{kl}}\boldsymbol{p} = \\ \gamma\cdot\mathrm{diag}(\beta-\boldsymbol{p})\mathrm{diag}(\boldsymbol{I}^{ex})\boldsymbol{e}_l\cdot g(p_k)\,. \quad (62)\end{aligned}$$

When we define the term in curled brackets as $-\boldsymbol{\mathcal{L}}^T$, we obtain eqn. (45). Multiplication with $-\boldsymbol{\mathcal{L}}^{T^{-1}}$ results in eqn. (44).
